# A Bioinformatics Analysis Based on Omics and Clinical Data for Graph-Based Patient Stratification in Hepatocellular Carcinoma

**DOI:** 10.3390/biology15151287

**Published:** 2026-08-04

**Authors:** Paolo Pio Bevilacqua, Paola Paci, Giulia Fiscon

**Affiliations:** 1 Department of Computer Science, Sapienza University of Rome, 00185 Rome, Italy; bevilacqua.2002288@studenti.uniroma1.it; 2 Department of Computer, Control and Management Engineering, Sapienza University of Rome, 00185 Rome, Italy; paola.paci@uniroma1.it; 3 Institute for Systems Analysis and Computer Science “Antonio Ruberti”, National Research Council, 00185 Rome, Italy; 4 Department for the Promotion of Human Sciences and Quality of life, San Raffaele Open University, 00166 Rome, Italy

**Keywords:** hepatocellular carcinoma, multi-omics integration, similarity network fusion, patient stratification, precision medicine, disease subtyping, transcriptomics, miRNA

## Abstract

Hepatocellular carcinoma (HCC) is one of the most frequent and lethal malignancies worldwide, with substantial molecular and clinical heterogeneity that complicates prognostic assessment. We aimed to identify molecular subgroups with distinct prognostic profiles using a graph-based multi-omics approach. Similarity Network Fusion (SNF) was applied to integrate mRNA and miRNA sequencing data from the TCGA-LIHC cohort (366 patients), and spectral clustering on the fused similarity network was used to identify patient subgroups. Survival differences were assessed by log-rank test and Cox regression; subgroup characterisation combined direction-aware over-representation analysis and one-versus-all differential expression. Six clusters with distinct survival trajectories were identified in the discovery cohort (exploratory log-rank *p*-value = 3.6 × 10^−4^). C1 (24.3% of patients) showed the worst prognosis (median OS 22.6 months; 5-year OS 28.0%), while C4 reached the best outcomes (median OS not reached; HR 0.34, *p*-value = 0.001). Cluster membership retained independent prognostic value after adjustment for age, sex and AJCC stage. Each cluster was assigned a descriptive transcriptional label (proliferative–oncofoetal, xenobiotic-metabolising, imprinted/fetal-reactivated, hepatocyte-zonation-like, cholangio-mesenchymal, mature-hepatocyte). As independent evidence of prognostic relevance, the subtype structure was reproduced in a differently profiled external cohort (GSE14520; log-rank *p*-value = 0.011), with the poor-prognosis (C1) and favourable (C4) subtypes retaining their ordering. SNF-based integration provides a reproducible framework for prognostically informative patient stratification in HCC, with the molecular descriptors serving as starting points for downstream biological characterisation on independent cohorts.

## Simple Summary

Liver cancer is one of the most common and deadly cancers worldwide, and patients with this disease can have very different outcomes even when their tumours look similar under the microscope. This study aimed to find hidden patterns in liver tumours that could help doctors better predict how a patient’s disease might progress. We analysed genetic activity data from the tumours of 366 patients using a computational method that combines two types of molecular information into a single map of similarities between patients. This approach revealed six distinct groups of patients, each with a different biological profile and survival outlook, ranging from a group with the poorest prognosis to one with the best. Even after accounting for a patient’s age, sex, and how advanced their cancer was at diagnosis, the group a patient belonged to still gave useful information about their likely survival. These findings suggest that looking at the molecular make-up of a tumour, rather than just its appearance, could help identify patients who need more aggressive treatment or closer monitoring, moving liver cancer care closer to a personalised approach. Further studies in other groups of patients are needed before these findings can be used in the clinic.

## 1. Introduction

Hepatocellular carcinoma (HCC) is the most common form of primary liver malignancy, typically arising in the setting of chronic liver disease and cirrhosis. In 2023, an estimated 41,210 new cases were diagnosed in the United States, an incidence rate that has tripled over the past four decades. HCC incidence and mortality are two to three times higher in men than in women, regardless of disease aetiology [1]. The marked molecular and clinical heterogeneity of HCC represents a major challenge for accurate prognostic assessment and treatment selection, making integrated bioinformatics approaches indispensable for identifying patient subgroups with distinct prognostic and therapeutic profiles.

The precision medicine paradigm aims to categorise patients into subgroups based on shared biological characteristics, providing more effective individualised treatments for improved clinical outcomes [2]. Data integration has emerged as a powerful strategy for disease subtyping, as individual omics analyses are limited to identifying correlations that more accurately reflect reactive rather than causal processes [3]. By integrating multiple data types, such as mRNA and miRNA expression, complementary biological information can be leveraged to uncover complex patterns that single data modalities cannot reveal in isolation [4,5].

Among integrative approaches, Similarity Network Fusion (SNF) [6] has emerged as a particularly effective graph-based technique. SNF constructs separate patient similarity networks for each data type and iteratively fuses them into a unified network, capturing both shared and complementary information across modalities. Unlike simple concatenation methods, which may dilute biological signal with technical noise, or sequential analyses that often yield inconsistent findings, SNF preserves the intrinsic structure of each dataset while enabling cross-modal integration. Its network-based architecture confers robustness to noise, heterogeneous data types, and small sample sizes, making it well-suited for multi-omics patient stratification [6]. Network-based approaches more broadly have become a unifying framework in network medicine for integrating heterogeneous molecular data and translating it into clinically relevant insights [7,8].

Several molecular classification frameworks have been proposed to capture the heterogeneity of HCC. Early transcriptome-based studies identified distinct subclasses associated with specific genetic alterations, proliferation status and clinical outcome [9,10], and the Cancer Genome Atlas subsequently provided a comprehensive multi-platform characterisation integrating mutational, copy-number, methylation and expression data to define integrative molecular subtypes of HCC [11]. More recently, multi-omics data integration strategies, including similarity- and network-based fusion approaches, have been used to refine patient stratification beyond single-omics signatures, revealing subtypes with distinct prognostic and biological profiles [6]. A systematic benchmarking of multi-omics integration methods for cancer subtyping further supports the competitive performance of network-fusion approaches such as SNF relative to alternative integration strategies [12].

Beyond these classical fusion strategies, several graph-learning approaches have recently been proposed for multi-omics cancer subtyping. Comparative studies of graph-convolutional, graph-attention and graph-transformer architectures have benchmarked these models for multi-omics cancer classification, generally reporting gains over feature-concatenation baselines at the cost of greater architectural complexity [13]. Heterogeneous-graph frameworks, such as HeteroGATomics, explicitly represent multiple node and edge types across omics layers and couple this representation with a joint feature-selection procedure, improving both integration performance and the interpretability of the resulting biomarkers [14]. Contrastive-learning frameworks, including multi-view multi-level contrastive graph convolutional networks, learn discriminative omics-specific and fused representations by enforcing embedding consistency, achieving competitive subtyping performance across a large panel of TCGA cohorts [15]. Anchor- and bipartite-graph formulations have likewise been introduced to reduce the computational cost of graph construction and to yield more cluster-friendly representations for multi-omics clustering [16]. More recent developments further illustrate this landscape as follows: heterogeneous-graph and anchor-graph multi-omics clustering (MOH [17]; scAGCI [18]) and graph contrastive-learning and deep graph-clustering strategies (GCIRS [19]; scGDCF [20]); some of these operate at the single-cell or spatial level rather than the patient level and are therefore noted here as related methodological directions rather than direct competitors to the present patient-level analysis. These graph-learning methods can in principle capture non-linear and higher-order relationships beyond those accessible to similarity-based fusion; however, they typically require larger training cohorts, extensive hyperparameter and architecture tuning, and generally offer more limited interpretability than classical network-fusion approaches. We selected SNF for the present study because it is a well-established, deterministic and computationally efficient graph-fusion method that requires no training procedure, has demonstrated robustness in small-to-moderate sample sizes such as the TCGA-LIHC cohort analysed here, and yields a patient similarity network that is directly interpretable and amenable to downstream survival and functional analyses. We regard the graph-learning approaches discussed above as complementary directions that may become increasingly attractive as larger and better-annotated HCC multi-omics cohorts become available.

**Figure 1:**
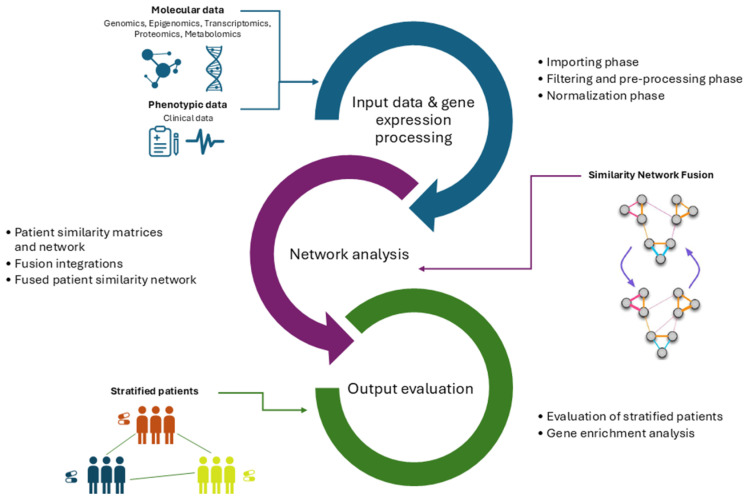
Workflow of the implemented pipeline.

The present work exploited the SNF framework to integrate mRNA and miRNA expression data from the TCGA dataset of Liver Hepatocellular Carcinoma cohort to identify clinically relevant patient subgroups characterised by distinct molecular and prognostic profiles. We complemented the clustering analysis with comprehensive survival analyses, differential expression profiling, functional enrichment, and clinical associations to provide a complete characterisation of the identified subtypes (Figure 1). Our approach aimed to demonstrate that molecular stratification provides prognostic information beyond that captured by conventional clinical staging alone, representing a step towards precision oncology in liver cancer.

## 2. Materials and Methods

### 2.1. Data Retrieval

The TCGA-LIHC dataset was obtained from the Cancer Genome Atlas (TCGA) project through the Genomic Data Commons (GDCs) portal [7]. The dataset comprises mRNA expression profiles (60,600 genes from 374 tumour and 50 normal samples) and miRNA expression profiles (1046 miRNAs from 375 tumour and 51 normal samples). Clinical data were extracted from the standard TCGA-LIHC clinical file and the curated Synapse data resource [21]. The variables retained for cluster-level testing were age at diagnosis, sex, AJCC pathologic stage (and individual T/N/M components), vital status, follow-up time, pharmacological and radiation treatment indicators, and prior malignancy. Aetiological annotations (HBV/HCV serology, alcohol consumption, NAFLD/NASH, BMI, cirrhosis, and family history of malignancy) were available only for a fraction of the cohort (missing rates between 18% and 78% depending on the variable) and were therefore not included in the cross-cluster comparison.

The mRNA matrix was obtained as continuous FPKM-like normalised expression values (versioned Ensembl gene identifiers) and the miRNA matrix as reads-per-million-miRNA-mapped (RPM) values; both were used after a log_2_(x + 1) transformation only, without further re-normalisation.

### 2.2. Data Preprocessing

For mRNA data, tumour and normal samples were identified using TCGA barcodes, and paired tumour–normal cases were isolated. Genes with zero overall expression were removed, and a log_2_(x + 1) transformation was applied to stabilise variance. Feature selection was performed using the Interquartile Range (IQR), with genes below the 64th percentile threshold removed. Differential expression analysis was conducted on 50 matched tumour–normal pairs using a paired Student’s t-test, with Benjamini–Hochberg correction (adjusted *p*-value < 0.01), yielding 14,033 differentially expressed genes. The final mRNA expression matrix retained all 371 tumour samples. For patients with more than one tumour aliquot, the first aliquot per 12-character TCGA patient barcode was retained and additional aliquots were discarded (no averaging), reducing 374 tumour samples to 371 unique patients.

For miRNA data, only tumour samples were retained (372 unique patients after duplicate removal). miRNAs with zero mean expression were removed (~18% of features), leaving 852 miRNAs. A log_2_(x + 1) transformation was applied. No differential expression analysis was performed on miRNAs, as their primary role was to provide complementary regulatory information for multi-omics integration. Following independent preprocessing, both matrices were harmonised to 366 common patients. The same first-aliquot-per-patient rule reduced 375 miRNA tumour samples to 372 unique patients.

We note that the mRNA and miRNA feature-selection strategies were not symmetric: mRNA features were filtered using paired tumour–normal differential expression, whereas miRNA features were filtered only by removing near-zero-expression transcripts, without an equivalent differential-expression step. This asymmetry reflects the structure of the available data rather than an arbitrary methodological choice: paired tumour–normal samples, which are required to perform a paired differential-expression test, were available for a substantial subset of mRNA profiles but not for the miRNA dataset, in which essentially only tumour samples were profiled. Applying an unpaired or single-group differential-expression criterion to the miRNA data was considered less reliable given the smaller and less balanced sample composition, so a conservative variance-/expression-based filter was preferred to retain a broader, less assumption-dependent panel of miRNAs for the fusion step. We acknowledge, however, that this asymmetry could in principle bias the relative contribution of each omics layer to the fused similarity network and, consequently, the discovered clusters. To assess this, we performed a sensitivity analysis in which mRNA and miRNA features were selected using matched, symmetric criteria (highly variable features selected via the Interquartile Range for both platforms, without relying on paired differential expression), and re-ran the full SNF and clustering pipeline on the resulting feature sets.

Under a symmetric, Interquartile-Range-based selection applied identically to both platforms (20,329 mRNA and 307 miRNA features, without paired differential expression), the pipeline again recovered six clusters that were moderately to substantially concordant with the original solution (adjusted Rand index = 0.67), with meaningful changes in individual patient assignments, and retained an overall-survival separation (log-rank *p*-value = 2.2 × 10^−3^) on the same patients. This indicates that part of the clustering structure and its prognostic signal persist under an alternative preprocessing strategy; because the survival separation is obtained from the same patients, it does not constitute independent confirmation. A cross-tabulation of cluster reassignment under the two feature-selection strategies is provided in Table S2. Detailed results are reported in Table S2.

### 2.3. Similarity Network Fusion

Patient stratification was performed using the SNF algorithm [6] via the SNFtool R package [6]. Both expression matrices were standardised using z-score normalisation. Patient-to-patient distances were calculated using Spearman correlation-based distance (1 − ρ), preferred for its robustness to outliers and ability to capture monotonic relationships. From each distance matrix, a patient affinity matrix was constructed using a scaled exponential kernel. The SNF algorithm then iteratively updated each network through a diffusion-like process, reinforcing cross-validated patient relationships. The following three hyperparameters were optimised via grid search: K (nearest neighbours) ∈ {10, 15, 20, 50, 60, 80, 100, 120}, α (kernel width) ∈ {0.4, 0.6, 0.8, 1.0, 1.2}, and T (iterations) ∈ {20, 25, 30, 50, 100, 200}. Optimal parameters were K = 10, α = 1.0, and T = 20, selected by a composite score incorporating silhouette width, separation ratio, NMI, and cluster balance.

### 2.4. Cluster Identification and Quality Assessment

Spectral clustering was applied to the fused similarity matrix. To characterise the choice of the number of clusters, we evaluated the eigen-gap of the normalised graph Laplacian, the average silhouette width, and a composite cluster-quality score (defined below) for alternative cluster numbers (k = 2^–10^); these are reported in Table S1b. None of these internal criteria identified a sharp optimum at six clusters: the eigen-gap was maximal at k = 8 (second highest at k = 4), while the average silhouette width and the composite score increased monotonically up to k = 10. The six-subtype solution was therefore not selected based on internal clustering-quality metrics, which lacked a clear optimum. We explicitly acknowledge that the choice of six clusters was partly outcome-informed: among the candidate solutions that were internally comparable, six clusters were retained because they yielded biologically and clinically interpretable subgroups with distinct overall-survival trajectories and distinct transcriptional programmes. Consequently, the discovery-cohort log-rank separation should be regarded as exploratory, and the independent GSE14520 cohort provides the primary evidence for the prognostic relevance of the subtypes. Normalised mutual information between the individual omics networks (NMI = 0.22) was also assessed.

The composite quality score combined four components, computed for each candidate parameter configuration on the fused network as follows: the mean silhouette width s (on the distance 1 − W_fused, floored at 0), a separation ratio r (mean within-cluster similarity divided by mean between-cluster similarity), the normalised mutual information m between the fused clustering and the individual mRNA and miRNA network clusterings, and a cluster-balance term b (one minus the coefficient of variation in the cluster sizes). The score was defined as Q = 0.4·s + 0.3·(r/r_max) + 0.2·m + 0.1·b, where the separation ratio and the balance term were scaled relative to their maximum across the parameter grid, while the silhouette width and NMI, which are already bounded in [0, 1], entered unscaled. The weights (0.4, 0.3, 0.2, 0.1) prioritise internal cohesion/separation over network concordance and cluster balance.

Survival data were not used in SNF hyperparameter tuning: the SNF parameters (K, α, and T) were chosen solely on the composite silhouette/separation/NMI/balance score and the eigen-gap heuristic described above. As noted above, however, survival separation did inform the selection of the number of clusters among internally comparable solutions; the discovery-cohort survival analysis is therefore treated as exploratory throughout. To assess the robustness of the survival separation to this unsupervised parameter-selection procedure, we additionally evaluated the log-rank statistic across nearby parameter settings and alternative cluster numbers; results are reported in Table S1. To account for the multiplicity introduced by examining several clustering resolutions, we further computed a selection-adjusted permutation *p*-value: for each of 10,000 permutations of the survival labels, the maximum log-rank statistic across all 36 candidate cluster partitions (k = 2–10 of the final network plus the 27 inspected SNF configurations) was retained, and the observed six-cluster statistic was compared against this null distribution (selection-adjusted *p*-value = 0.017).

To assess the stability and reproducibility of the six-subtype solution, we performed a resampling analysis. The full SNF pipeline (standard normalisation, 1—Spearman correlation distance, affinity-matrix construction with K = 10 and α = 1, network fusion over T = 20 iterations, and spectral clustering into six groups) was re-run on 100 random subsamples, each retaining 80% of patients; for each resample, the adjusted Rand index (ARI) between the subsample partition and the full-cohort reference labels was computed on the sampled patients. A consensus (co-clustering) matrix was accumulated across resamples, and per-subtype reproducibility was summarised as the mean within-cluster co-clustering proportion. To assess whether six clusters are better supported than alternative resolutions, comparable subsampling-based stability was also computed for each candidate number of clusters (k = 2–10), summarising the mean ARI across 100 subsamples at every k (mean ARI at k = 6 = 0.839; full profile in Table S6). Sensitivity to the two main hyper-parameters was assessed by re-running the full-cohort clustering across grids of the neighbourhood size (K ∈ {6, 8, 10, 12, 15, 20, 25, 30}) and the number of fusion iterations (T ∈ {10, 15, 20, 25, 30, 50}), reporting the ARI against the reference solution in each case. Analyses used the SNFtool and mclust R packages (Supplementary Figure S8, Supplementary Table S6).

### 2.5. Survival Analysis

Overall survival (OS) was defined as time from diagnosis to death or last follow-up. Kaplan–Meier survival curves were estimated for each cluster, and differences were evaluated using the log-rank test [22]. Univariate and multivariate Cox proportional hazards regression models [23] were employed to quantify hazard ratios (HRs) with 95% confidence intervals, adjusting for age at diagnosis, sex, and AJCC pathologic stage. The proportional hazards assumption was tested using Schoenfeld residuals.

### 2.6. Post-Clustering Molecular Characterisation

Differential expression analysis was performed using the limma package with empirical Bayes moderated t-statistics in a one-versus-all comparison design. For visualisation, the union of the top up-regulated markers per cluster was assembled into a subtype signature and displayed as a gene-wise z-score-standardised heatmap, with genes and patients block-ordered by cluster assignment (generated by the project pipeline). Significance thresholds were adjusted *p*-value < 0.01 with |log_2_FC| > 1 for mRNA and |log_2_FC| > 0.5 for miRNA. Functional enrichment analysis was conducted using over-representation analysis (ORA) via the clusterProfiler R package, querying Gene Ontology Biological Process (GO BP) and KEGG pathway databases. Clinical associations were assessed using the Kruskal–Wallis test (continuous variables) and Pearson’s chi-square test (categorical variables), with Benjamini–Hochberg correction. To avoid direction-agnostic results, ORA was performed separately on the up- and down-regulated DEG lists; enrichment terms in the main text are reported with the direction of their driver genes. The top-ranked up- and down-regulated genes obtained from this one-versus-all analysis are reported per cluster in Appendix A.

### 2.7. Tumour Purity and Immune/Stromal Composition Analysis

Because bulk transcriptomic clusters can in principle be driven by differences in sample composition rather than by malignant-cell-intrinsic biology alone, we examined tumour purity and immune/stromal content across the six SNF-derived clusters, treating composition as a potential co-determinant of the cluster profiles rather than a factor to be excluded. Purity and immune and stromal scores available for the TCGA-LIHC cohort (e.g., ESTIMATE-derived ImmuneScore, StromalScore and tumour purity) were retrieved and compared across clusters using the Kruskal–Wallis test with Benjamini–Hochberg correction. This analysis was intended to clarify whether cluster-specific transcriptional programmes reflect intrinsic tumour-cell biology, differences in non-malignant cell infiltration, or a combination of both.

### 2.8. Benchmarking Against Established HCC Molecular Subtypes

To clarify the relationship between our SNF-derived clusters and previously pub-lished HCC molecular classifications, we benchmarked cluster assignments against representative transcriptional signatures and, where available, genomic features from established subtyping schemes. Published subtype signatures were obtained programmatically from the Molecular Signatures Database (MSigDB), the Hoshida S1/S2/S3 classes, Boyault subgroup signatures, a Chiang CTNNB1/Wnt-β-catenin activation signature, a Hallmark inflammatory-response signature used as a proxy for immune activation, and a Yamashita liver stem-cell (progenitor/cholangiocytic-like) signature and were never hand-transcribed; their provenance is recorded together with the analysis code. Each tumour was scored for every signature by single-sample gene-set enrichment analysis (ssGSEA; GSVA package) applied to our expression data and assigned to the corresponding class. Somatic mutation calls for TP53 and CTNNB1 were obtained from the TCGA-LIHC mutation annotation file downloaded from the Genomic Data Commons (TCGAbiolinks). Association between cluster membership and each published subtype or mutation category was tested using Fisher’s exact test or a Monte-Carlo approximation of Fisher’s test where exact computation was intractable on sparse contingency tables, with Benjamini–Hochberg correction for multiple comparisons. TCGA integrative (iCluster) subtypes were not included, as a version of sample-to-subtype mapping was not available for this cohort.

### 2.9. External Validation in an Independent Cohort

To assess whether the six SNF-derived subtypes generalise beyond the TCGA-LIHC discovery cohort, we validated the clustering structure in an independent HCC dataset profiled on a different platform. We used the GSE14520 cohort (Roessler et al. [24]; Affymetrix HT Human Genome U133A array, GPL3921), which provides tumour gene-expression profiles together with overall-survival (OS) and recurrence-free-survival (RFS) follow-up.

Because the discovery pipeline integrates two omics layers that are not both available in GSE14520, and because external cohorts cannot be re-clustered de novo without redefining the subtypes, we transferred the TCGA-derived structure to the external cohort using a nearest-centroid classifier built exclusively from mRNA. From the one-versus-all differential-expression results (Section 2.6), we defined a transcriptional signature as the union of the top 50 up-regulated markers of each cluster (ranked by adjusted *p*-value and |log_2_FC|; duplicate symbols resolved by retaining the most significant), yielding 299 unique genes. TCGA cluster centroids were computed on this signature after gene-wise z-score standardisation, averaging the standardised expression of the discovery samples within each cluster. GSE14520 tumour samples were normalised identically (probe-to-gene collapse retaining the highest-variance probe per HGNC symbol, followed by gene-wise z-score standardisation) and each tumour was assigned to the cluster whose centroid showed the highest Spearman correlation across the shared signature genes. Euclidean distance was used as a sensitivity check, and the classification margin (difference between the top two correlations) was recorded for each sample.

Survival in the external cohort was analysed exactly as in the discovery cohort (Section 2.5): Kaplan–Meier curves with the log-rank test for the six predicted subtypes, and univariate and multivariate Cox proportional-hazards regression. The multivariate model adjusted for the covariates available in GSE14520 (BCLC stage, sex, age and cirrhosis); the C1 subtype, which showed the worst prognosis in the discovery cohort, was used as the reference category. To confirm that the cross-platform gene overlap did not drive the result, we repeated the entire procedure using a signature restricted a priori to the top 50 up-regulated markers of each cluster that are present on the GPL3921 array (293 genes, 100% cohort coverage; Supplementary Table S5). RFS was analysed as a second, independent survival endpoint. Analyses were performed in R using the survival package.

### 2.10. Software and Reproducibility

All analyses were performed in R 4.5.2 (31 October 2025). The main pipeline used SNFtool 2.3.1 for network fusion, limma 3.66.0 and edgeR 4.8.2 for differential expression, cluster 2.1.8.2 and mclust 6.1.2 for clustering quality (adjusted Rand index and normalised mutual information), survival 3.8.6 and survminer 0.5.2 for survival analysis, clusterProfiler 4.18.4 for functional enrichment, ComplexHeatmap 2.26.1 and ggplot2 4.0.2 for visualisation, and GEOquery 2.78.0 for the external cohort. The supplementary analyses additionally used GSVA 2.4.9 and msigdbr 26.1.0 (subtype signature scoring), estimate 1.0.13 (tumour purity/immune-stromal scores), FSA 0.10.1 (Dunn post hoc test), and TCGAbiolinks 2.38.0 (somatic mutation retrieval). A single random seed (123) was set before every stochastic step, network fusion, spectral clustering, silhouette evaluation, bootstrap resampling, grid search and cross-validation, to ensure full reproducibility. Somatic mutation calls for the benchmarking analysis were obtained from the GDC (Data Release 45.0, 4 December 2025; “Masked Somatic Mutation”, MAF consensus workflow; downloaded 13 July 2026). The mRNA and miRNA expression matrices were taken from the project data release rather than re-downloaded, and the corresponding GDC acquisition date is reported in the Data Availability statement.

## 3. Results

### 3.1. SNF-Based Patient Stratification

**Figure 2:**
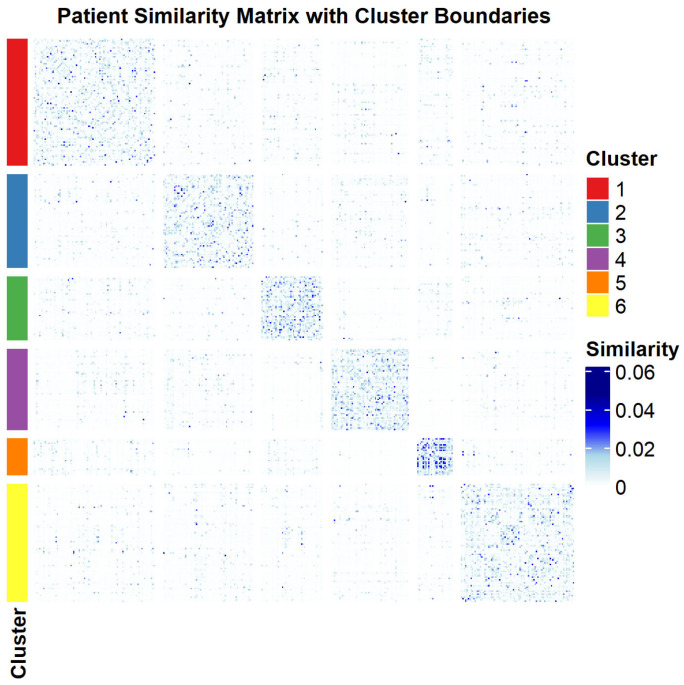
Heatmap of patient-to-patient similarity after network fusion, ordered by cluster assignment.

SNF integration of mRNA and miRNA expression profiles from 366 TCGA-LIHC patients, followed by spectral clustering, identified six molecular subtypes designated C1 through C6. The clusters contained 89 (24.3%), 66 (18.0%), 45 (12.3%), 57 (15.6%), 26 (7.1%), and 83 (22.7%) patients, respectively. Visual inspection of the fused similarity matrix ordered by cluster assignment revealed clearly delineated blocks of higher within-cluster similarity along the diagonal (Figure 2). Intra-cluster connections exhibited systematically higher similarity values than inter-cluster connections, confirming effective patient stratification.

The six-subtype partition was highly reproducible. Across 100 subsamples retaining 80% of patients, the mean adjusted Rand index against the full-cohort solution was 0.839 (median 0.840; range 0.760–0.840; and standard deviation 0.008), indicating good stability of the partition (rather than near-identical recovery). All six subtypes were individually stable, with mean within-cluster co-clustering proportions between 0.69 and 0.94; C5 showed the highest co-clustering proportion, which supports its cohesion conditional on the six-cluster solution but does not by itself establish it as a uniquely defined biological subtype. The solution was also robust to the choice of hyper-parameters: the ARI against the reference exceeded 0.85 for neighbourhood sizes K between 8 and 15 and remained between 0.82 and 0.94 across fusion iterations T from 10 to 50, confirming that the selected values (K = 10, T = 20) lie within a stable plateau rather than at a sharp optimum (Supplementary Figure S8, Supplementary Table S6). The prognostic separation was likewise stable to these choices: across a grid of 27 parameter settings and alternative cluster numbers, the six-cluster solution reproduced the reference partition at the selected setting (K = 10, T = 20; adjusted Rand index = 1.00) and every six-cluster configuration retained a significant global log-rank test (*p*-value between 1.7 × 10^−4^ and 5.2 × 10^−3^; Table S1).

### 3.2. Survival Analysis

**Figure 3:**
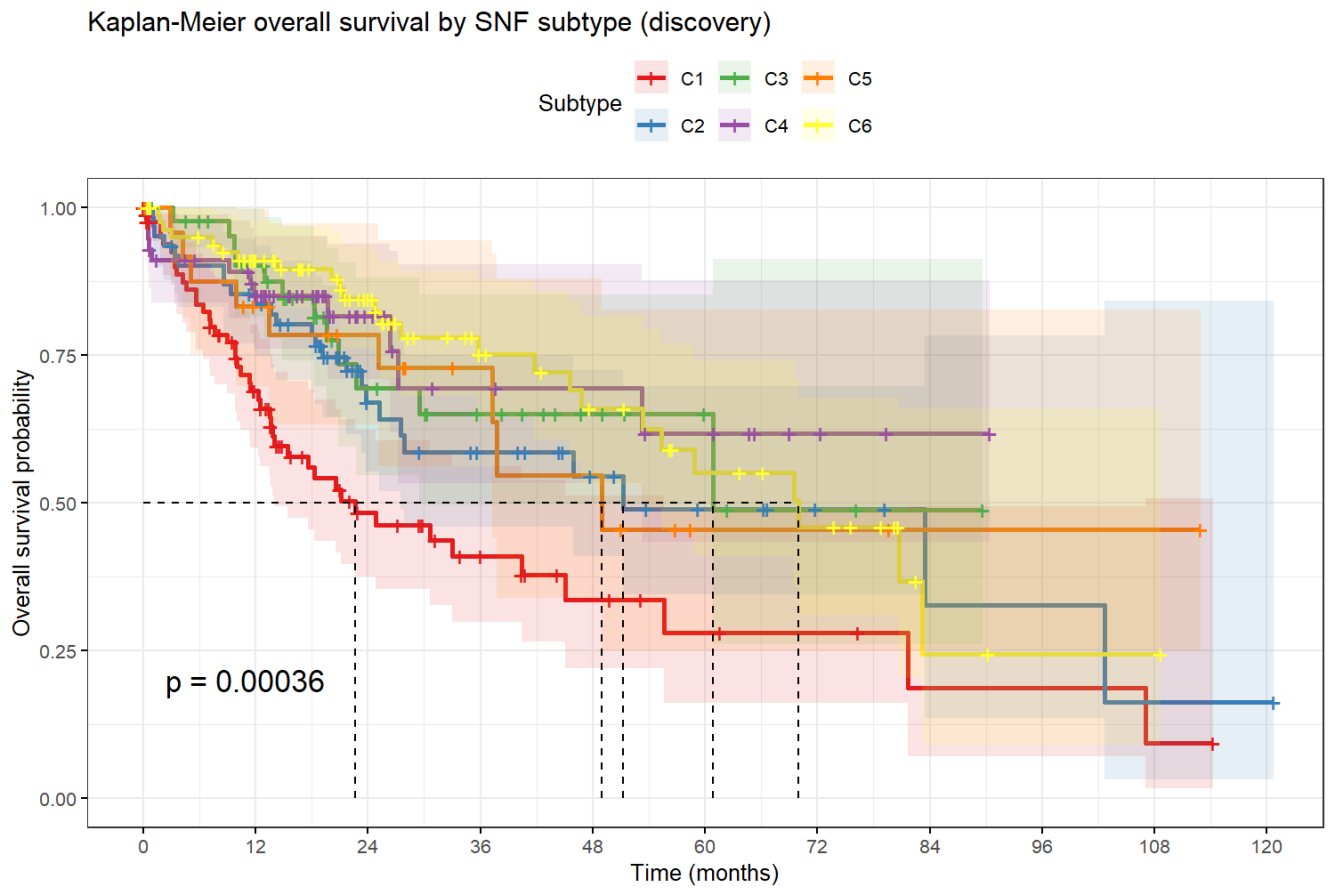
Kaplan–Meier survival curves of the SNF clusters.

The final survival cohort comprised 359 patients (127 death events, 35.4%; median follow-up: 21.4 months). The global log-rank test comparing survival across all six clusters yielded a highly significant result (χ^2^ = 22.8, df = 5, exploratory *p*-value = 3.6 × 10^−4^). Because the number of clusters was partly outcome-informed (Section 2.4), this discovery-cohort separation is interpreted as exploratory; a selection-adjusted permutation *p*-value of 0.017 is reported in Section 2.4, and the independent external cohort (Section 3.9) provides the primary prognostic evidence. Kaplan–Meier analysis (Figure 3) revealed marked prognostic differences: C1 exhibited the worst survival (median OS: 22.6 months; 5-year OS: 28.0%), while C4 showed the best outcomes (median OS not reached; 5-year OS: 61.7%). C6 also demonstrated favourable prognosis (median OS: 70.0 months; 5-year OS: 55.1%). Survival estimates at landmark time points are summarised in Table 1.

Univariate Cox regression (Table 1) confirmed that all subtypes exhibited significantly reduced hazard compared to C1, with HRs ranging from 0.36 (C4) to 0.54 (C2). Multivariate Cox regression adjusting for age, sex, and AJCC stage (Table 2) demonstrated that cluster membership retained independent prognostic significance. C2 (HR = 0.47, *p*-value = 0.004), C4 (HR = 0.34, *p*-value = 0.001), C5 (HR = 0.38, *p*-value = 0.010), and C6 (HR = 0.36, *p*-value < 0.001) remained significantly associated with improved survival. C3 showed marginal significance (HR = 0.52, *p*-value = 0.061), suggesting partial confounding by tumour stage. Among clinical covariates, AJCC Stage III (HR = 2.80, *p*-value < 0.001) and Stage IV (HR = 28.10, *p*-value = 0.002) were strongly associated with increased mortality, while age and sex were not independently significant. The proportional hazard assumption was satisfied (Schoenfeld global test *p*-value = 0.26). In terms of effective sample size, the univariate Cox models included all 359 patients of the survival cohort (127 events), whereas the multivariate model, after complete-case handling of the adjustment covariates, included 356 patients (125 events; three patients excluded for missing covariate values, two of them with an event).

### 3.3. Molecular Characterisation of HCC Subtypes

Differential expression analysis identified between 208 and 969 DEGs per cluster, with substantial variation in both number and directional bias (Table 3). The most striking finding was C5, which exhibited 969 DEGs, approximately 2.7- to 4.7-fold more than any other cluster, despite being the smallest subgroup (*n* = 26). C1 and C4 showed predominant downregulation (59% and 58%, respectively), whereas C5 and C6 demonstrated strong upregulation bias (67% and 73%). miRNA analysis revealed the following complementary regulatory patterns: C4 exhibited massive global downregulation (86% of 193 DEMs), while C1 and C3 showed strong miRNA upregulation (85% each). The ten most strongly up-regulated and the ten most strongly down-regulated genes of each cluster are listed in Table A1 (Appendix A).

**Figure 4:**
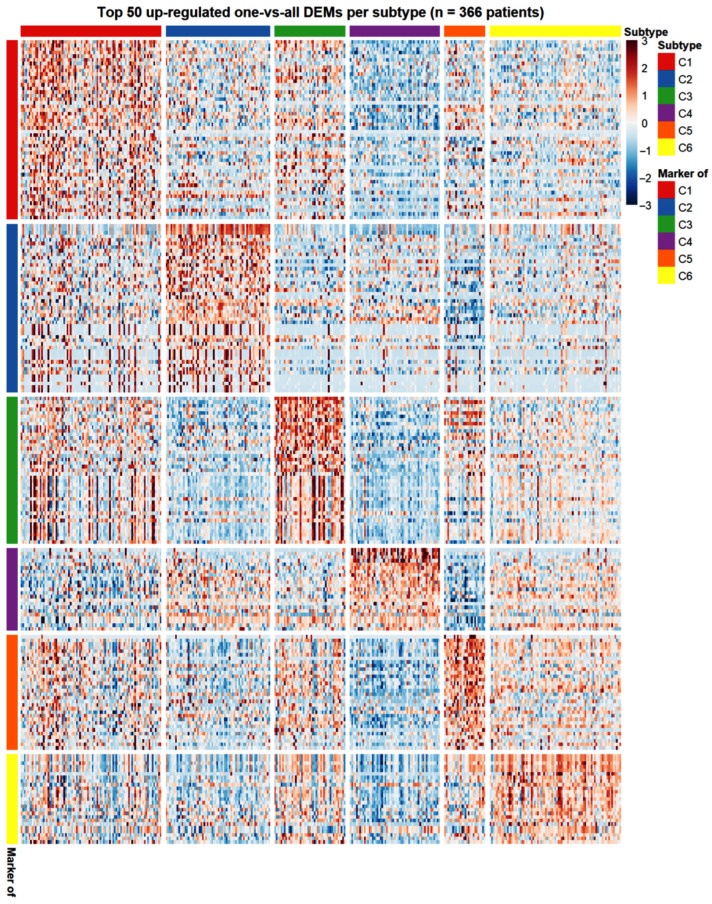
Heatmap of the 299-gene subtype signature across the 366 TCGA-LIHC tumours. Rows are the union of the top up-regulated one-versus-all markers of each subtype (approximately 50 per cluster; duplicates resolved by significance) and columns are patients; both are ordered by cluster assignment (C1–C6), with white gaps separating the six blocks. Expression is gene-wise z-scored (row-standardised) and capped at ±3.

The cluster-specific expression structure underlying these subtypes is shown in Figure 4, which displays the 299-gene transcriptional signature (the union of the top up-regulated markers of each subtype) across all 366 patients, with genes and patients ordered by cluster assignment. Each subtype is characterised by a coherent block of up-regulated signature genes, producing the block-diagonal pattern expected of well-separated transcriptional programmes and visually corroborating the one-versus-all differential-expression results.

### 3.4. Functional Enrichment Analysis

**Figure 5:**
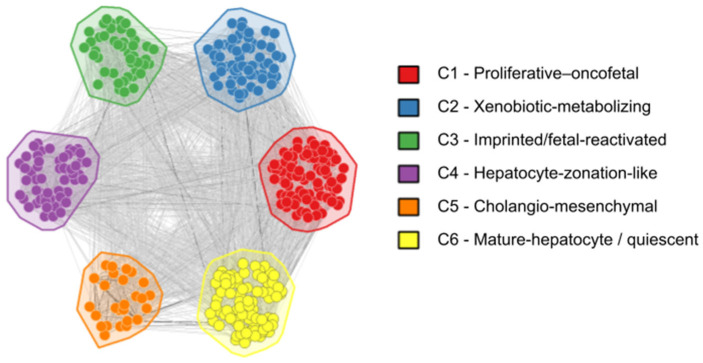
Patient similarity network with proposed cluster’s identity label.

Direction-aware GO BP enrichment confirmed both shared and cluster-specific signatures (Supplementary Figures S1–S6). CYP-mediated xenobiotic and retinol metabolism terms were enriched across most clusters but with opposite directionality: in C2, C4 and C6 they were driven by up-regulated genes (preserved or enhanced hepatocyte programmes), while in C1 and C5 they were driven by down-regulated genes (coordinated loss of hepatocyte programmes). Cluster-specific signatures included up-regulation-driven enrichment for steroid metabolism in C3, and enrichment for the GO BP term “immunoglobulin-mediated immune response” in C4, the latter being driven by 37 down-regulated immunoglobulin genes (i.e., reduced rather than increased B-cell/plasma cell infiltrate). KEGG analysis complemented these findings: C5 showed up-regulation-driven enrichment for integrin signalling (consistent with ITGB4/ITGA3 up-regulation) and down-regulation-driven enrichment for bile secretion (loss of hepatocyte transporters such as ABCB11 and SLC10A1); C6 showed up-regulation-driven enrichment for complement and coagulation cascades and down-regulation-driven enrichment for cell-cycle terms; C2 and C3 showed up-regulation-driven enrichment for chemical carcinogenesis—DNA adducts (driven by phase-I/-II detoxification genes). Together, these pathway-level findings support the identification of six transcriptionally distinct HCC clusters with distinct prognostic profiles (Figure 5 and Figures S1–S6).

### 3.5. Subtype Characterisation

Based on the results of functional enrichment analysis, we assigned each cluster a descriptive label that summarises its dominant transcriptional features (Table 4); these labels are descriptive and are not intended as biological subtype assignments.

- **C1 (Proliferative–oncofetal).** C1 is the largest cluster (*n* = 89) and shows the poorest prognosis, with predominantly down-regulated mRNA (59% of DEGs) and strongly UP-skewed miRNA (85% of DEMs). Up-regulated genes combine a mitotic core (UBE2S, UBE2C, CDC20, and BIRC5) with oncofetal markers (AFP, S100P, SPINK1, and G6PD); on the miRNA side, oncogenic miR-18a and miR-210 are up-regulated and the miR-99a/100~let-7c~miR-125b cluster is down-regulated. Enrichment for hepatic metabolic terms is largely driven by down-regulated genes, reflecting loss of hepatocyte programmes (Supplementary Figure S1).
- **C2 (Xenobiotic-metabolising).** C2 (*n* = 66) shows intermediate survival and a transcriptional profile dominated by phase-I/-II detoxification genes (CYP2A6/2A7, CYP8B1, HSD17B13), with CYP2E1 notably down-regulated. The miR-216a/216b/217 locus is among the top up-regulated miRNAs of the cohort, while miR-200 family members are down-regulated; canonical EMT transcripts are not significantly up-regulated, so the miR-200 effect is not interpreted as an active EMT phenotype. Aetiological variables were not available, precluding a direct link to specific exposures (Supplementary Figure S2).
- **C3 (Imprinted/foetal-reactivated).** C3 (*n* = 45) shows favourable prognosis (5-year OS 65.1%) and it is the youngest cluster (mean age 50.5 years). Its dominant signal is reactivation of the H19/IGF2 imprinted locus: H19, AFP, GPC3, PEG10 and the foetal CYP3A7 isoform are among the top up-regulated transcripts, and miR-483 (logFC +6.26, the largest miRNA effect of the cohort) and miR-675 are the top up-regulated miRNAs. Stem-cell markers MSI1 and ARID3A are also up-regulated (Supplementary Figure S3).
- **C4 (Hepatocyte-zonation-like).** C4 (*n* = 57) shows the best prognosis (HR 0.34) and a profile dominated by zone-3-like hepatocyte markers: CYP3A4 (top up), CYP2E1, CYP1A2, GLUL, AXIN2, NKD1 and FBXO31 are all up-regulated, while the mitotic core and oncofetal panel (AFP, GPC3, and KRT19) are coordinately repressed. miRNA expression is globally down-regulated (86% of DEMs), with miR-122 preserved. The GO BP “immunoglobulin-mediated immune response” term is driven by 37 down-regulated immunoglobulin genes, indicating reduced rather than increased B-cell/plasma cell infiltrate in this cluster (Supplementary Figure S4).
- **C5 (Cholangio-mesenchymal).** C5 (*n* = 26) is the smallest and most transcriptionally distinctive cluster, with 969 DEGs (~2.7- to 4.7-fold more than any other). Up-regulated genes include cholangiocyte/stem-like markers (KRT19, SPP1, CD24, and SOX9), extracellular-matrix components (COL1A1/2, POSTN, BGN, and TGFB1/2), integrins (ITGB4, ITGA3) and glycolytic genes (PKM, PFKP, and HK2). Hepatocyte programmes are broadly repressed, and miR-122 shows the strongest negative effect of the cluster. Despite the most adverse stage distribution, median OS is intermediate (n = 26 limit inference) (Supplementary Figure S5).
- **C6 (Mature-hepatocyte/quiescent).** C6 (*n* = 83) shows the longest median OS (70.0 mo). Its profile combines coordinated up-regulation of acute-phase reactants (SAA1/2, CRP, and HAMP), coagulation (FGA/B/G, F9, and PLG) and complement components (C7, C9), CYP450 isoforms and urea-cycle enzymes (ARG1, OTC, and CPS1), with coordinated repression of >20 cell-cycle regulators (MCM3/5/7, CDT1, PTTG1, BIRC5, CCNB1, and MYBL2). The miR-199a/b family and additional tumour-suppressive miRNAs (miR-145, miR-139, and miR-150) are up-regulated, consistent with preserved hepatic synthetic function and reduced proliferative activity (Supplementary Figure S6).

### 3.6. Clinical Associations

After Benjamini–Hochberg correction, the following two clinical variables were significantly associated with cluster membership: age at diagnosis (Kruskal–Wallis adjusted *p*-value = 5.09 × 10^−6^) and sex (chi-square adjusted *p*-value = 1.44 × 10^−3^). Distribution of AJCC stage was uneven across clusters (chi-square adjusted *p*-value = 0.07 for stage group and adjusted *p*-value = 0.002 for the T component), with C5 showing the lowest proportion of stage I cases (15.4%). No other clinical variable reached statistical significance after correction. Aetiological variables were not tested due to high missing rates (see Section 2.1).

### 3.7. Tumour Purity and Immune/Stromal Composition

Tumour purity and ESTIMATE-derived immune/stromal scores were compared across the six clusters to assess whether cluster-specific transcriptional programmes could be partly explained by differences in non-malignant cell content rather than by malignant-cell-intrinsic biology alone. All four ESTIMATE-derived measures differed markedly across the six clusters (Kruskal–Wallis with Benjamini–Hochberg correction: StromalScore χ^2^ = 113.6, ImmuneScore χ^2^ = 81.0, ESTIMATEScore χ^2^ = 96.5 and tumour purity χ^2^ = 96.5, all df = 5, adjusted *p*-value < 1 × 10^−15^; Table S3). Composition varied systematically by subtype as follows: C4 showed the highest tumour purity (median 0.88) together with the lowest immune and stromal scores, whereas C1 and C6 showed the lowest purity and the highest immune/stromal content. Pairwise Dunn post hoc comparisons (Benjamini–Hochberg-corrected) are reported in Table S3b, and the full score distributions are shown in Figure S7. These results indicate that non-malignant cell content varies substantially across subtypes. The large between-cluster differences imply that tumour purity and immune/stromal infiltration may be important drivers of the clustering; the cluster profiles should therefore be interpreted as potentially reflecting a combination of malignant-cell-intrinsic programmes and sample-composition effects, rather than malignant-cell-intrinsic biology alone. While the composition differences are concordant with recognised HCC biology (e.g., an immune-high, stroma-rich phenotype), this concordance does not exclude a compositional contribution to the transcriptional signal, and the prognostic separation of the subtypes remained detectable in the complementary sensitivity analyses (Section 2.2 and Section 3.1). The strongest pairwise differences involved C4 and C6: tumour purity separated C4 from C6 (adjusted *p*-value = 1.5 × 10^−14^) and C6 from C2 (9.4 × 10^−13^), stromal content most strongly distinguished C4 from C6 (7.9 × 10^−17^) and C2 from C6 (9.5 × 10^−17^), and immune score most strongly separated C1 from C4 (6.2 × 10^−10^) and C4 from C6 (9.4 × 10^−10^), consistent with the high-purity, immune/stroma-low profile of C4 and the opposite profile of C6.

### 3.8. Benchmarking Against Established HCC Molecular Subtypes

Cluster assignments were compared against the Hoshida, Boyault, TCGA integrative (iCluster), CTNNB1/Wnt-β-catenin, immune-class and KRT19+/EpCAM+ progenitor-/cholangiocytic-like classification schemes, as described in Section 2.8. Every scheme was significantly associated with cluster membership (Benjamini–Hochberg-adjusted *p*-value < 0.01 throughout). The Hoshida differentiated class (S3) dominated C6, C2 and C4 (≥95% of patients), whereas the proliferation class (S2) was concentrated in C3 and the aggressive class (S1) was confined to C5 (Monte-Carlo Fisher *p*-value < 1 × 10^−5^). Boyault subgroup signatures behaved concordantly, with the G1 signature unique to C3 and the G6 signature enriched in C4 (68%; *p*-value < 1 × 10^−5^). The Wnt/β-catenin activation signature was highest in C4 (91%) and C6 (72%) and absent from C5 (Fisher *p*-value = 2.4 × 10^−22^), mirroring the CTNNB1 mutation pattern. The inflammatory/immune-activation signature was highest in C6 (83%), C5 (73%) and C1 (57%) and lowest in C4 (14%, immune-cold; Fisher *p*-value = 7.6 × 10^−19^); this ordering coincided with the ESTIMATE ImmuneScore ranking obtained independently in Section 3.7, providing internal cross-validation of the immune-hot subtypes. The pro-genitor/cholangiocytic-like signature was highest in C5 (85%), C3 (82%) and C6 (65%) and lowest in C4 (14%; Fisher *p*-value = 8.8 × 10^−17^). At the genomic level, TP53 mutations were enriched in C2 (47%) and C1 (44%) and rare in C4 (9%; Fisher *p*-value = 6.3 × 10^−8^), whereas CTNNB1 mutations were strongly concentrated in C4 (72%) and rare in C3 and C5 (4%; Fisher *p*-value = 3.3 × 10^−17^). Taken together, C4 emerges as the canonical CTNNB1-mutant, Wnt-activated, immune-cold, well-differentiated subtype, C1 and C2 as the TP53-enriched aggressive subtypes, and C3/C5 as progenitor/stem-like; TCGA iCluster labels were not available for this cohort and were therefore not benchmarked.

### 3.9. External Validation in an Independent Cohort

The 299-gene nearest-centroid classifier assigned all 225 GSE14520 tumours to one of the six subtypes; 221 tumours had OS follow-up (85 death events, 38.5%; median follow-up 57 months) and entered the survival analysis. Of the 299 signature genes, 174 (58.2%) were represented on the HT_HG-U133A array. Spearman- and Euclidean-based assignments agreed for 85.8% of tumours.

**Figure 6:**
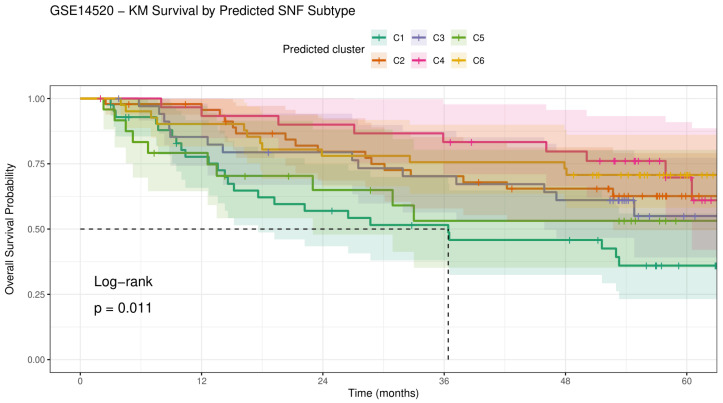
Kaplan–Meier overall-survival curves for the six SNF-derived subtypes predicted in the independent GSE14520 cohort.

The six predicted subtypes showed significantly different overall survival in this fully independent, differently profiled cohort (log-rank χ^2^ = 14.9, df = 5, *p*-value = 0.011; Figure 6, Table 5). The prognostic ordering observed in the discovery cohort was preserved: C1 (Proliferative–oncofetal) again showed the poorest outcome and was the only subtype to reach its median OS (36.4 months; event rate 55.8%), whereas C4 (Hepatocyte-zonation-like) again showed the most favourable outcome (median OS not reached; event rate 29.0%). Univariate Cox regression confirmed that C1 carried the highest hazard: relative to C1, C4 had an HR of 0.34 (95% CI 0.16–0.73, *p*-value = 0.006), C6 an HR of 0.36 (0.18–0.71, *p*-value = 0.004) and C2 an HR of 0.45 (0.24–0.86, *p*-value = 0.015). Treating C1 versus all other subtypes as a single contrast, patients assigned to C1 had more than twice the mortality hazard of the remaining cohort (HR = 2.20, 95% CI 1.37–3.54, *p*-value = 0.0011).

In a multivariate Cox model adjusting for BCLC stage, sex, age and cirrhosis (219 tumours, 84 events), subtype membership retained independent prognostic value: C4 remained significantly associated with improved survival relative to C1 (HR = 0.38, 95% CI 0.17–0.84, *p*-value = 0.016), with the remaining subtypes showing HRs below 1 in the same direction as the discovery cohort. As expected, advanced BCLC stage was the dominant clinical predictor (stage C HR = 4.50, 95% CI 2.53–8.00, *p*-value < 0.001), while age and sex were not independently significant. The prognostic signal was reproduced as a second endpoint: for RFS, C1 again carried the highest recurrence hazard (C1 versus rest HR = 1.84, 95% CI 1.22–2.79, *p*-value = 0.004), although the six-group RFS log-rank test reached only a trend level (*p*-value = 0.097).

Two sensitivity analyses confirmed that the validation was not an artefact of the cross-platform gene loss. First, restricting the signature a priori to the 293 markers present on the GPL3921 array (100% cohort coverage, thereby removing the 58.2% overlap entirely) reproduced the same result (log-rank *p*-value = 0.018; C1 versus rest HR = 2.15, 95% CI 1.32–3.50, *p*-value = 0.002; C4 multivariate HR = 0.38, *p*-value = 0.016), with 88.9% of tumours receiving the same label as under the full signature (Supplementary Table S5). Second, the C1-versus-rest hazard ratio was stable across classification-confidence subsets (HR 2.15–2.36; all *p*-value < 0.05), indicating that lower-confidence assignments did not inflate the effect. Together, these analyses demonstrate that the SNF-derived subtype structure, and particularly the C1/C4 prognostic extremes, generalises to an independent cohort profiled on a different transcriptomic platform.

## 4. Discussion

In this study, SNF-based integration of mRNA and miRNA expression profiles identified six molecular subtypes of hepatocellular carcinoma with markedly different survival trajectories. This result reinforces the view that integrating complementary omics layers can reveal clinically relevant structure that is not apparent from single-platform analyses, consistent with the rationale that motivated the adoption of network-based data fusion in the present work [3,4,5,6]. Related network-based multi-omics strategies have similarly proven effective at uncovering disease-relevant molecular determinants in other complex disorders, such as chronic obstructive pulmonary disease [25], further supporting the value of network-level integration across different pathological contexts. Importantly, cluster membership retained independent prognostic value after adjustment for age, sex and AJCC stage, indicating that the molecular subtypes capture prognostic information that is complementary to, rather than redundant with, conventional clinical staging.

The subtype structure proved robust to patient resampling and to the choice of SNF hyper-parameters (mean ARI = 0.839 under 80% subsampling; stable across K ∈ [8, 15] and T ∈ [10, 50]), indicating that the six-group solution reflects reproducible structure in the data rather than an artefact of a particular parameter configuration.

The six subtypes span a biological continuum, from clusters dominated by proliferative and oncofetal reactivation programmes (C1, C3) to clusters characterised by preserved or enhanced mature hepatocyte function (C4, C6), with an intermediate xenobiotic-metabolising profile (C2) and a transcriptionally distinct cholangio-mesenchymal subtype (C5) marked by extracellular-matrix remodelling and loss of hepatocyte identity. This gradient mirrors the concept of hepatocellular dedifferentiation as a driver of aggressive disease and is consistent with the poorer prognosis observed for clusters in which oncofetal and mitotic programmes predominate over mature liver function. The direction-aware functional enrichment results further support this interpretation, linking the same pathways to opposite outcomes depending on whether they were driven by up- or down-regulated genes.

Positioned against established HCC molecular classification axes, several of our clusters show plausible qualitative correspondence. C1 and C3, dominated by proliferative/mitotic and oncofetal markers (AFP, GPC3, and mitotic-core genes) and, for C3, reactivation of the H19/IGF2 imprinted locus, align conceptually with the proliferation-class tumours described by Hoshida and colleagues and with the more proliferative Boyault subgroups, which are similarly enriched for AFP expression and progenitor-like features. C5, characterised by KRT19, SPP1 and CD24 up-regulation together with extracellular-matrix remodelling and loss of mature hepatocyte identity, closely parallels the previously described KRT19+/EpCAM+ progenitor-like, cholangiocarcinoma-like subtype that is consistently associated with poor prognosis across independent HCC cohorts. Conversely, C4 and C6, marked by preserved or enhanced CYP450 and urea-cycle gene expression and by the best survival outcomes, are consistent with the well-differentiated, non-proliferative tumours that in previous classifications were enriched for CTNNB1/Wnt-β-catenin pathway activation. C2 occupies an intermediate, xenobiotic-metabolism-dominated position that does not map as clearly onto a single published axis. These qualitative correspondences were confirmed by the formal benchmarking (Section 3.8, Table S4): cluster membership was significantly associated with every published signature scheme tested and with TP53 and CTNNB1 mutation status. In particular, the CTNNB1/Wnt-activated, immune-cold profile of C4 was corroborated at the genomic level (72% CTNNB1-mutant, only 9% TP53-mutant), the TP53-enriched C1 and C2 matched their aggressive transcriptional assignments, and the progenitor/stem-like character of C3 and C5 was reflected in both the stem-cell signature and the Hoshida proliferation/aggressive classes; the immune-hot ordering agreed with the ESTIMATE-based composition analysis, providing convergent evidence from independent methods. The following two scope limitations remain: the immune axis was represented by an inflammatory-response signature rather than a dedicated validated immune-class classifier, and TCGA integrative (iCluster) labels were not available for this cohort; these two comparisons therefore remain to be confirmed with dedicated signatures.

From a translational perspective, these findings suggest that a network-based multi-omics classification could complement AJCC staging in identifying patients with more aggressive disease trajectories who might benefit from closer surveillance or more intensive treatment. The reproducibility of the SNF pipeline and its modest data requirements (two omics layers, with no additional experimental assays) make it a practical candidate for further evaluation in prospective or independent retrospective cohorts. Moreover, network-based frameworks originally developed for drug repurposing [26], including applications to human cancers [27], could in principle be coupled with the present molecular subtypes to prioritise candidate therapies tailored to each HCC subtype. The reproducibility of the prognostic ordering in an independent microarray cohort, obtained with a compact mRNA-only classifier, indicates that the clinically most relevant axis of the classification, the separation between the proliferative–oncofetal (C1) and the well-differentiated hepatocytic (C4/C6) extremes, is transferable across platforms and does not depend on the miRNA layer used at the discovery stage.

We also acknowledge that, as with any bulk-transcriptomic classification, the clusters identified here may reflect a mixture of malignant-cell-intrinsic transcriptional programmes and sample-composition effects, including differences in tumour purity and immune or stromal cell infiltration; our tumour-purity and immune/stromal composition analysis (Section 3.7) was designed to explore this possibility. Beyond composition, several clinical and biological variables that are known or suspected to influence HCC biology and prognosis, disease aetiology (e.g., viral hepatitis, alcohol-related liver disease, NAFLD/NASH), presence and severity of cirrhosis, underlying liver function, prior treatment, vascular invasion, and batch or technical effects related to sample processing, could not be comprehensively assessed in the present cohort because of missing or incomplete annotation (see Section 2.1) or because such information is not systematically captured in the TCGA-LIHC resource. As a result, we cannot fully exclude residual confounding by these factors, and the biological interpretation of the six clusters should be regarded as hypothesis-generating rather than definitive until it can be re-examined in cohorts with more complete clinical, aetiological and pathological annotation.

**Limitations.** This study has several limitations, including reliance on a single discovery cohort of matched miRNA and mRNA data (TCGA-LIHC), a predominantly Western patient composition, and the inclusion of only two omics layers. We validated the subtype structure in one independent, differently profiled HCC cohort (GSE14520, microarray) using an mRNA-only nearest-centroid classifier, and the prognostic separation and the C1/C4 ordering were reproduced (log-rank *p*-value = 0.011); nonetheless, the validation was necessarily transcriptome-only and cross-platform, the association was weaker than in the discovery cohort (*p*-value = 0.011 versus 3.6 × 10^−4^), and confirmation in prospective and non-Western cohorts, ideally with matched multi-omics profiling, is still required before clinical translation can be considered. In addition, several potential confounders—tumour aetiology, liver function, cirrhosis severity, treatment, vascular invasion, and technical or batch effects—could not be fully adjusted for given the available data and high missingness; the cluster profiles may therefore partly reflect these factors together with tumour purity and immune/stromal composition, and the biological and clinical claims should be interpreted with corresponding caution. Integrating additional data types (DNA methylation, somatic mutations, and proteomics) could further refine subtype definitions and strengthen their biological interpretation.

## 5. Conclusions

We applied Similarity Network Fusion to the joint analysis of mRNA and miRNA expression profiles of 366 TCGA-LIHC patients and obtained six clusters with different overall-survival trajectories in the discovery cohort (exploratory log-rank *p*-value = 3.6 × 10^−4^), whose prognostic relevance was independently supported in an external cohort (GSE14520; log-rank *p*-value = 0.011). Cluster membership retained independent prognostic value after adjustment for age, sex and AJCC stage in multivariate Cox regression (HR 0.34–0.52 vs. C1, all *p*-value ≤ 0.05). Post-clustering analysis described each cluster in terms of the dominant up- and down-regulated programmes and direction-aware pathway enrichment. The contribution of the present study is providing a reproducible, network-based pipeline for HCC patient stratification, together with the molecular descriptors of the six clusters, which are intended as a starting point for downstream biological characterisation on independent cohorts.

## Figures and Tables

**Table 1:** Kaplan–Meier survival estimates by molecular subtype.

Cluster	N	Events	Median OS (mo)	5-yr OS (%)	HR (Univariate)	*p*-Value
C1	84	44	22.6	28.0	1.00 (ref)	—
C2	66	25	51.2	49.0	0.54	0.016
C3	44	12	60.8	65.1	0.41	0.006
C4	57	12	NR	61.7	0.36	0.002
C5	26	9	48.9	45.5	0.48	0.043
C6	83	25	70.0	55.1	0.38	<0.001

Abbreviations: N = number of patients; OS = overall survival; HR = hazard ratio; NR = not reached; mo = months.

**Table 2:** Multivariate Cox proportional hazards regression results.

Variable	HR	95% CI	*p*-Value
Cluster (ref: C1)			
C2	0.47	0.28–0.79	0.004
C3	0.52	0.27–1.03	0.061
C4	0.34	0.18–0.66	0.001
C5	0.38	0.18–0.79	0.010
C6	0.36	0.21–0.61	<0.001
Age at diagnosis	1.00	1.00–1.00	0.280
Sex: male (ref: female)	0.95	0.64–1.39	0.786
Stage II (ref: Stage I)	1.40	0.83–2.35	0.203
Stage III	2.80	1.79–4.36	<0.001
Stage IV	28.10	3.58–220.88	0.002

**Table 3:** Summary of differentially expressed genes (DEGs) and miRNAs (DEMs) per cluster.

Cluster	Total DEGs	Up (%)	Down (%)	Total DEMs	DEM up (%)	DEM down (%)
C1	262	108 (41)	154 (59)	117	99 (85)	18 (15)
C2	208	128 (62)	80 (38)	168	88 (52)	80 (48)
C3	211	107 (51)	104 (49)	115	98 (85)	17 (15)
C4	352	147 (42)	205 (58)	193	27 (14)	166 (86)
C5	969	653 (67)	316 (33)	103	44 (43)	59 (57)
C6	216	157 (73)	59 (27)	67	42 (63)	25 (37)

Thresholds: mRNA adjusted *p*-value < 0.01, |log_2_FC| > 1; miRNA adjusted *p*-value < 0.01, |log_2_FC| > 0.5. Abbreviations: DEMs = differentially expressed miRNAs.

**Table 4:** Integrated molecular and clinical characteristics of the six HCC subtypes. Arrows indicate direction of regulation; ↑ up-regulation, ↓ downregulation.

Cluster	Median OS (mo)	5-yr OS (%)	Key Molecular Signature	Proposed Identity
C1 (24.3%)	22.6	28.0	mRNA ↓; miRNA ↑; mitotic core + oncofetal markers; hepatocyte-loss	Proliferative–oncofetal
C2 (18.0%)	51.2	49.0	Balanced mRNA/miRNA; xenobiotic response, CYP450	Xenobiotic-metabolising
C3 (12.3%)	60.8	65.1	Balanced mRNA; miRNA ↑; H19/IGF2 reactivation; oncofetal markers	Imprinted/foetal-reactivated
C4 (15.6%)	NR	61.7	mRNA ↓; miRNA ↓↓ (86%); Zone-3-like CYPs + GLUL; mitotic core down	Hepatocyte-zonation-like
C5 (7.1%)	48.9	45.5	969 DEGs; KRT19+/SPP1+; ECM/TGFβ; hepatic-loss; miR-122 down	Cholangio-mesenchymal
C6 (22.7%)	70.0	55.1	mRNA ↑↑; complement/coagulation; Cell cycle ↓	Mature-hepatocyte/ quiescent

**Table 5:** External validation of the SNF subtypes in the GSE14520 cohort (*n* = 221).

Subtype (Label)	N	Events	Median OS (mo)	Event Rate	Univariate HR vs. C1 (95% CI)	*p*-Value
C1 (Proliferative–oncofetal)	43	24	36.4	55.8%	1.00 (ref)	—
C2 (Xenobiotic-metabolising)	47	16	NR	34.0%	0.45 (0.24–0.86)	0.015
C3 (Imprinted/foetal-reactivated)	35	14	NR	40.0%	0.55 (0.28–1.06)	0.072
C4 (Hepatocyte-zonation-like)	31	9	NR	29.0%	0.34 (0.16–0.73)	0.006
C5 (Cholangio-mesenchymal)	24	10	NR	41.7%	0.74 (0.35–1.55)	0.424
C6 (Mature-hepatocyte)	41	12	NR	29.3%	0.36 (0.18–0.71)	0.004

Global log-rank test across the six subtypes: χ^2^ = 14.9, df = 5, *p*-value = 0.011. C1 versus all other subtypes combined: HR = 2.20 (95% CI 1.37–3.54), *p*-value = 0.0011. Abbreviations: N = number of patients; OS = overall survival; HR = hazard ratio; NR = not reached; mo = months; ref = reference category.

## Data Availability

The results shown here are based upon data generated by the TCGA Research Network: https://www.cancer.gov/tcga, accessed on 27 January 2026. The developed pipeline is available at https://github.com/ppbevilacqua/hcc-snf-molecular-subtypes, accessed on 27 January 2026. The mRNA and miRNA expression matrices and the associated clinical data for the TCGA-LIHC cohort were downloaded from the GDC data portal on 27 January 2026.

## Supplementary Materials

The following supporting information can be downloaded at https://www.mdpi.com/article/10.3390/biology15151287/s1, Figure S1: Direction-aware Gene Ontology Biological Process enrichment for cluster C1 (Proliferative–oncofoetal); Figure S2: Direction-aware Gene Ontology Biological Process enrichment for cluster C2 (Xenobiotic-metabolising); Figure S3: Direction-aware Gene Ontology Biological Process enrichment for cluster C3 (Imprinted/foetal-reactivated); Figure S4: Direction-aware Gene Ontology Biological Process enrichment for cluster C4 (Hepatocyte-zonation-like); Figure S5: Direction-aware Gene Ontology Biological Process enrichment for cluster C5 (Cholangio-mesenchymal); Figure S6: Direction-aware Gene Ontology Biological Process enrichment for cluster C6 (Mature-hepatocyte/quiescent); Figure S7: Distribution of tumour purity and ESTIMATE immune/stromal scores across the six clusters; Figure S8: Cluster robustness analysis (resampling ARI, per-subtype consensus, and K/T hyper-parameter sensitivity); Table S1: Stability of the cluster–survival association across nearby SNF hyperparameter settings and alternative numbers of clusters; Table S1b: Selection of the number of clusters; Table S2: Sensitivity analysis of clustering and survival results to a matched, symmetric mRNA/miRNA feature-selection strategy; Table S3: Kruskal–Wallis comparison of tumour purity and immune/stromal scores across clusters; Table S3b: Dunn’s post-hoc pairwise comparisons between subtypes (Benjamini-Hochberg adjusted) for each of the four ESTIMATE-derived scores; Table S4: Benchmarking of the six clusters against established HCC molecular subtype schemes (Hoshida, Boyault, TCGA iCluster, CTNNB1/Wnt, immune class, KRT19+/EpCAM+ progenitor-like) and TP53/CTNNB1 mutation status. Table S5: External-validation sensitivity analysis in GSE14520 using an array-restricted signature (293 genes, 100% platform coverage), reporting concordance with the full 299-gene classifier and the corresponding survival statistics. Table S6: Numerical summary of the cluster robustness analysis: subsampling ARI, per-subtype co-clustering consensus, hyper-parameter (K/T) sensitivity, and subsampling stability across candidate cluster numbers (k = 2–10).

## Appendix A. Top 10 Differentially Expressed Genes per Cluster

For each of the six SNF-derived clusters identified in the TCGA-LIHC cohort, the ten most significantly upregulated and the ten most significantly downregulated genes are reported. Differential expression analysis was performed using a one-versus-all approach, comparing each cluster against all remaining samples. Genes are ranked by ascending adjusted *p*-value (FDR correction, Benjamini–Hochberg method) within each direction of regulation. For each gene, the Ensembl identifier with its corresponding HGNC symbol, the log_2_ fold-change (logFC), and the adjusted *p*-value (adjusted *p*-value) are provided. Only genes passing the significance threshold (adjusted *p*-value < 0.01 and |logFC| > 1) were considered for inclusion.

**Table A1:** Top 10 differentially expressed genes per cluster.

Cluster	Gene	Log FC	Adjusted *p*-Value
C1	*ENSG00000163993.7|S100P*	2.742078	2.10 × 10^−12^
	*ENSG00000081051.8|AFP*	2.594533	7.48 × 10^−11^
	*ENSG00000164266.11|SPINK1*	2.372947	5.07 × 10^−8^
	*ENSG00000110492.15|MDK*	1.952122	7.52 × 10^−19^
	*ENSG00000118785.14|SPP1*	1.916727	2.97 × 10^−6^
	*ENSG00000181019.13|NQO1*	1.878879	3.48 × 10^−8^
	*ENSG00000211896.7|IGHG1*	1.842084	1.84 × 10^−5^
	*ENSG00000101057.16|MYBL2*	1.743407	4.31 × 10^−20^
	*ENSG00000211592.8|IGKC*	1.728556	5.22 × 10^−5^
	*ENSG00000160211.19|G6PD*	1.720542	2.44 × 10^−24^
	*ENSG00000198099.9|ADH4*	−2.3373	2.51 × 10^−10^
	*ENSG00000138115.14|CYP2C8*	−2.378752	7.38 × 10^−17^
	*ENSG00000170509.12|HSD17B13*	−2.38164	2.84 × 10^−12^
	*ENSG00000175003.15|SLC22A1*	−2.47382	2.28 × 10^−13^
	*ENSG00000180432.6|CYP8B1*	−2.502616	4.91 × 10^−14^
	*ENSG00000198650.11|TAT*	−2.720981	3.67 × 10^−16^
	*ENSG00000100652.5|SLC10A1*	−2.810715	2.96 × 10^−20^
	*ENSG00000117594.10|HSD11B1*	−2.857828	4.26 × 10^−16^
	*ENSG00000255974.8|CYP2A6*	−3.071351	4.23 × 10^−13^
	*ENSG00000160868.15|CYP3A4*	−3.341388	2.55 × 10^−14^
C2	*ENSG00000255974.8|CYP2A6*	3.886745	9.99 × 10^−16^
	*ENSG00000170509.12|HSD17B13*	3.236258	5.48 × 10^−17^
	*ENSG00000198077.10|CYP2A7*	2.852576	1.51 × 10^−17^
	*ENSG00000175003.15|SLC22A1*	2.641404	2.53 × 10^−11^
	*ENSG00000244067.3|GSTA2*	2.590842	6.92 × 10^−10^
	*ENSG00000151365.2|THRSP*	2.567501	7.25 × 10^−11^
	*ENSG00000198650.11|TAT*	2.538084	1.69 × 10^−10^
	*ENSG00000180432.6|CYP8B1*	2.513453	1.47 × 10^−10^
	*ENSG00000256612.7|CYP2B7P*	2.355136	7.88 × 10^−15^
	*ENSG00000126752.8|SSX1*	2.329700	1.62 × 10^−15^
	*ENSG00000160932.11|LY6E*	−1.610567	9.82 × 10^−9^
	*ENSG00000211895.5|IGHA1*	−1.675215	4.51 × 10^−6^
	*ENSG00000211896.7|IGHG1*	−1.721349	8.80 × 10^−4^
	*ENSG00000114115.10|RBP1*	−1.746312	1.03 × 10^−10^
	*ENSG00000137077.8|CCL21*	−1.799693	5.84 × 10^−8^
	*ENSG00000211592.8|IGKC*	−1.897929	1.85 × 10^−4^
	*ENSG00000088992.18|TESC*	−1.978561	1.05 × 10^−12^
	*ENSG00000090382.7|LYZ*	−2.015775	9.17 × 10^−9^
	*ENSG00000148346.12|LCN2*	−2.607489	5.27 × 10^−10^
	*ENSG00000130649.10|CYP2E1*	−2.811550	1.35 × 10^−8^
C3	*ENSG00000130600.19|H19*	3.560017	7.45 × 10^−13^
	*ENSG00000081051.8|AFP*	3.396319	2.53 × 10^−10^
	*ENSG00000147257.15|GPC3*	3.175111	5.14 × 10^−11^
	*ENSG00000242265.6|PEG10*	3.064217	2.57 × 10^−12^
	*ENSG00000271824.2|SMIM32*	2.248543	4.71 × 10^−22^
	*ENSG00000133134.12|BEX2*	2.120825	1.62 × 10^−12^
	*ENSG00000068366.20|ACSL4*	2.103259	2.18 × 10^−9^
	*ENSG00000127831.11|VIL1*	2.077466	1.66 × 10^−13^
	*ENSG00000078898.7|BPIFB2*	2.052176	6.33 × 10^−11^
	*ENSG00000160870.15|CYP3A7*	1.897080	3.44 × 10^−9^
	*ENSG00000118785.14|SPP1*	−2.138333	1.38 × 10^−4^
	*ENSG00000166741.8|NNMT*	−2.139630	3.57 × 10^−7^
	*ENSG00000158104.11|HPD*	−2.222698	1.69 × 10^−5^
	*ENSG00000151365.2|THRSP*	−2.226239	1.77 ×10^−6^
	*ENSG00000151632.17|AKR1C2*	−2.232874	6.91 × 10^−13^
	*ENSG00000117594.10|HSD11B1*	−2.317721	2.79 × 10^−6^
	*ENSG00000113600.11|C9*	−2.332762	4.96 × 10^−6^
	*ENSG00000180432.6|CYP8B1*	−2.380616	2.33 × 10^−7^
	*ENSG00000188257.12|PLA2G2A*	−2.557863	2.65 × 10^−5^
	*ENSG00000173432.12|SAA1*	−2.875577	7.44 × 10^−7^
C4	*ENSG00000160868.15|CYP3A4*	4.951208	1.54 × 10^−22^
	*ENSG00000108602.18|ALDH3A1*	3.867853	3.59 × 10^−29^
	*ENSG00000130649.10|CYP2E1*	3.621758	5.31 × 10^−13^
	*ENSG00000135821.19|GLUL*	3.350701	4.97 × 10^−36^
	*ENSG00000165376.11|CLDN2*	3.289843	1.74 × 10^−27^
	*ENSG00000103569.10|AQP9*	3.275756	2.20 × 10^−21^
	*ENSG00000140505.7|CYP1A2*	3.213316	2.61 × 10^−19^
	*ENSG00000117594.10|HSD11B1*	3.143505	2.23 × 10^−13^
	*ENSG00000172016.16|REG3A*	3.141528	3.03 × 10^−11^
	*ENSG00000101951.17|PAGE4*	3.053637	7.86 × 10^−29^
	*ENSG00000130600.19|H19*	−2.348966	4.06 × 10^−7^
	*ENSG00000173511.10|VEGFB*	−2.350197	1.19 × 10^−29^
	*ENSG00000163993.7|S100P*	−2.586340	1.07 × 10^−7^
	*ENSG00000211592.8|IGKC*	−2.602369	4.18 × 10^−7^
	*ENSG00000084110.11|HAL*	−2.733949	5.81 × 10^−20^
	*ENSG00000112299.8|VNN1*	−2.790693	7.12 × 10^−20^
	*ENSG00000272398.6|CD24*	−2.958247	8.47 × 10^−19^
	*ENSG00000177459.11|ERICH5*	−3.013504	4.35 × 10^−26^
	*ENSG00000164266.11|SPINK1*	−3.210315	1.04 × 10^−9^
	*ENSG00000132693.12|CRP*	−3.246513	6.05 × 10^−9^
C5	*ENSG00000171345.13|KRT19*	4.228568	2.72 × 10^−25^
	*ENSG00000118785.14|SPP1*	3.511869	1.86 × 10^−7^
	*ENSG00000146038.12|DCDC2*	3.210977	3.96 × 10^−23^
	*ENSG00000088992.18|TESC*	3.029887	1.82 × 10^−14^
	*ENSG00000067225.18|PKM*	2.862389	8.23 × 10^−21^
	*ENSG00000128242.13|GAL3ST1*	2.804911	3.07 × 10^−17^
	*ENSG00000132470.14|ITGB4*	2.804113	8.80 × 10^−40^
	*ENSG00000272398.6|CD24*	2.785681	7.38 × 10^−9^
	*ENSG00000235162.9|C12orf75*	2.778849	1.22 × 10^−22^
	*ENSG00000163191.6|S100A11*	2.678167	5.09 × 10^−18^
	*ENSG00000129596.5|CDO1*	−3.545498	1.40 × 10^−25^
	*ENSG00000243955.6|GSTA1*	−3.602205	3.45 × 10^−12^
	*ENSG00000118271.12|TTR*	−3.628219	4.34 × 10^−15^
	*ENSG00000257017.9|HP*	−3.673207	5.27 × 10^−14^
	*ENSG00000255974.8|CYP2A6*	−3.719764	2.37 × 10^−7^
	*ENSG00000103569.10|AQP9*	−3.951259	8.95 × 10^−16^
	*ENSG00000110245.12|APOC3*	−4.031733	9.77 × 10^−19^
	*ENSG00000105398.4|SULT2A1*	−4.100079	7.24 × 10^−19^
	*ENSG00000118137.10|APOA1*	−4.127062	3.74 × 10^−16^
	*ENSG00000145192.13|AHSG*	−4.659669	1.05 × 10^−20^
C6	*ENSG00000188257.12|PLA2G2A*	3.225274	5.03 × 10^−13^
	*ENSG00000173432.12|SAA1*	3.212975	8.15 × 10^−14^
	*ENSG00000113600.11|C9*	2.949366	3.50 × 10^−15^
	*ENSG00000135094.11|SDS*	2.849582	1.44 × 10^−13^
	*ENSG00000132693.12|CRP*	2.667505	1.26 × 10^−8^
	*ENSG00000134339.9|SAA2*	2.591599	1.12 × 10^−15^
	*ENSG00000112936.19|C7*	2.547199	1.58 × 10^−27^
	*ENSG00000105697.9|HAMP*	2.357106	7.30 × 10^−19^
	*ENSG00000198650.11|TAT*	2.332128	2.27 × 10^−11^
	*ENSG00000255974.8|CYP2A6*	2.299417	2.15 × 10^−7^
	*ENSG00000131747.15|TOP2A*	−1.396090	1.11 × 10^−16^
	*ENSG00000167900.12|TK1*	−1.418487	2.56 × 10^−21^
	*ENSG00000237649.8|KIFC1*	−1.454224	6.87 × 10^−23^
	*ENSG00000089685.15|BIRC5*	−1.506283	2.01 × 10^−21^
	*ENSG00000164611.13|PTTG1*	−1.556241	2.63 × 10^−22^
	*ENSG00000175063.17|UBE2C*	−1.595182	7.32 × 10^−18^
	*ENSG00000117399.14|CDC20*	−1.605729	1.20 × 10^−18^
	*ENSG00000101057.16|MYBL2*	−1.653369	5.88 × 10^−17^
	*ENSG00000242265.6|PEG10*	−1.883254	2.80 × 10^−8^
	*ENSG00000081051.8|AFP*	−2.206680	8.85 × 10^−8^

## References

[1] Sung H., Ferlay J., Siegel R.L., Laversanne M., Soerjomataram I., Jemal A., Bray F. (2021). Global Cancer Statistics 2020: GLOBOCAN Estimates of Incidence and Mortality Worldwide for 36 Cancers in 185 Countries. CA A Cancer J. Clin..

[2] Wang R.C., Wang Z. (2023). Precision Medicine: Disease Subtyping and Tailored Treatment. Cancers.

[3] Hasin Y., Seldin M., Lusis A. (2017). Multi-Omics Approaches to Disease. Genome Biol..

[4] Subramanian I., Verma S., Kumar S., Jere A., Anamika K. (2020). Multi-Omics Data Integration, Interpretation, and Its Application. Bioinform. Biol. Insights.

[5] Ritchie M.D., Holzinger E.R., Li R., Pendergrass S.A., Kim D. (2015). Methods of Integrating Data to Uncover Genotype–Phenotype Interactions. Nat. Rev. Genet..

[6] Wang B., Mezlini A.M., Demir F., Fiume M., Tu Z., Brudno M., Haibe-Kains B., Goldenberg A. (2014). Similarity Network Fusion for Aggregating Data Types on a Genomic Scale. Nat. Methods.

[7] Fiscon G., Conte F., Farina L., Paci P. (2018). Network-Based Approaches to Explore Complex Biological Systems Towards Network Medicine. Genes.

[8] Silverman E.K., Schmidt H.H.H.W., Anastasiadou E., Altucci L., Angelini M., Badimon L., Balligand J.-L., Benincasa G., Capasso G., Conte F. (2020). Molecular Networks in Network Medicine: Development and Applications. Wiley Interdiscip. Rev. Syst. Biol. Med..

[9] Hoshida Y., Nijman S.M.B., Kobayashi M., Chan J.A., Brunet J.-P., Chiang D.Y., Villanueva A., Newell P., Ikeda K., Hashimoto M. (2009). Integrative Transcriptome Analysis Reveals Common Molecular Subclasses of Human Hepatocellular Carcinoma. Cancer Res..

[10] Boyault S., Rickman D.S., de Reynies A., Balabaud C., Rebouissou S., Jeannot E., Herault A., Saric J., Belghiti J., Franco D. (2007). Transcriptome Classification of HCC Is Related to Gene Alterations and to New Therapeutic Targets. Hepatology.

[11] Ally A., Balasundaram M., Carlsen R., Chuah E., Clarke A., Dhalla N., Holt R.A., Jones S.J.M., Lee D., Ma Y. (2017). Comprehensive and Integrative Genomic Characterization of Hepatocellular Carcinoma. Cell.

[12] Duan R., Gao L., Gao Y., Hu Y., Xu H., Huang M., Song K., Wang H., Dong Y., Jiang C. (2021). Evaluation and Comparison of Multi-Omics Data Integration Methods for Cancer Subtyping. PLoS Comput. Biol..

[13] Alharbi F., Vakanski A., Zhang B., Elbashir M.K., Mohammed M. (2025). Comparative Analysis of Multi-Omics Integration Using Graph Neural Networks for Cancer Classification. IEEE Access.

[14] Tabakhi S., Vandermeulen C., Sudbery I., Lu H. (2024). Heterogeneous Graph Attention Network Improves Cancer Multiomics Integration. arXiv.

[15] Yang B., Cui C., Wang M., Ji H., Gao F. (2025). Multi-View Multi-Level Contrastive Graph Convolutional Network for Cancer Subtyping on Multi-Omics Data. Brief. Bioinform..

[16] Zhu S., Liu H., Cui M. (2024). Efficient Multi-Omics Clustering with Bipartite Graph Subspace Learning for Cancer Subtype Prediction. Electron. Res. Arch..

[17] Dong Y., Chen C., Shi J., Hu Y., Cao X., Dong Y., Zhang X. (2026). MOH: A Novel Multilayer Multi-Omics Heterogeneous Graph for Single-Cell Clustering. IEEE J. Biomed. Health Inform..

[18] Dong Y., Zhang J., Shi J., Hu Y., Cao X., Dong Y., Zhang X. (2025). scAGCI: Anchor-Graph Contrastive Integration for Single-Cell Multi-Omics Clustering. Brief. Bioinform..

[19] Dong Y., Ai H., Yao S., Cao X., Li K., Dong Y., Zhang X. (2026). Graph Contrastive Learning for Inferring Spatial Cell Composition from Integrated Single-cell RNA Sequencing and Spatial Transcriptomics. IEEE J. Biomed. Health Inform..

[20] Dong Y., Li K., Dong Y., Ren Z., Zhang J., Hu Y., Zhang X. (2026). ScGDCF: Graphical Deep Clustering with Fused Common Information for Single-cell RNA-seq Data. IEEE Trans. Comput. Biol. Bioinform..

[21] Liu J., Lichtenberg T., Hoadley K.A., Poisson L.M., Lazar A.J., Cherniack A.D., Kovatich A.J., Benz C.C., Levine D.A., Lee A.V. (2018). An Integrated TCGA Pan-Cancer Clinical Data Resource to Drive High-Quality Survival Outcome Analytics. Cell.

[22] Mantel N. (1966). Evaluation of Survival Data and Two New Rank Order Statistics Arising in Its Consideration. Cancer Chemother. Rep..

[23] Cox D.R. (1972). Regression Models and Life-Tables. J. R. Stat. Soc. Ser. B Stat. Methodol..

[24] Roessler S., Jia H.-L., Budhu A., Forgues M., Ye Q.-H., Lee J.-S., Thorgeirsson S.S., Sun Z., Tang Z.-Y., Qin L.-X. (2010). A Unique Metastasis Gene Signature Enables Prediction of Tumor Relapse in Early-Stage Hepatocellular Carcinoma Patients. Cancer Res..

[25] Paci P., Fiscon G., Conte F., Licursi V., Morrow J., Hersh C., Cho M., Castaldi P., Glass K., Silverman E.K. (2020). Integrated Transcriptomic Correlation Network Analysis Identifies COPD Molecular Determinants. Sci. Rep..

[26] Fiscon G., Paci P. (2021). SAveRUNNER: An R-Based Tool for Drug Repurposing. BMC Bioinform..

[27] Galimi A., Fiscon G. (2025). A Network-Based Approach Exploiting Transcriptomics and Interactomics Data for Predicting Drug Repurposing Solutions Across Human Cancers. Cancers.

