# Supplementary material for: A Bioinformatics Analysis Based on Omics and Clinical Data for Graph-Based Patient Stratification in Hepatocellular Carcinoma
Source: Biology (Basel). 2026 Aug 4;15(15):1287. doi: 10.3390/biology15151287 (PMC13465779; doi:10.3390/biology15151287)

Figure S3. Subtype C3 − Imprinted / fetal−reactivated

GO Biological Process enrichment (up− vs down−regulated genes)

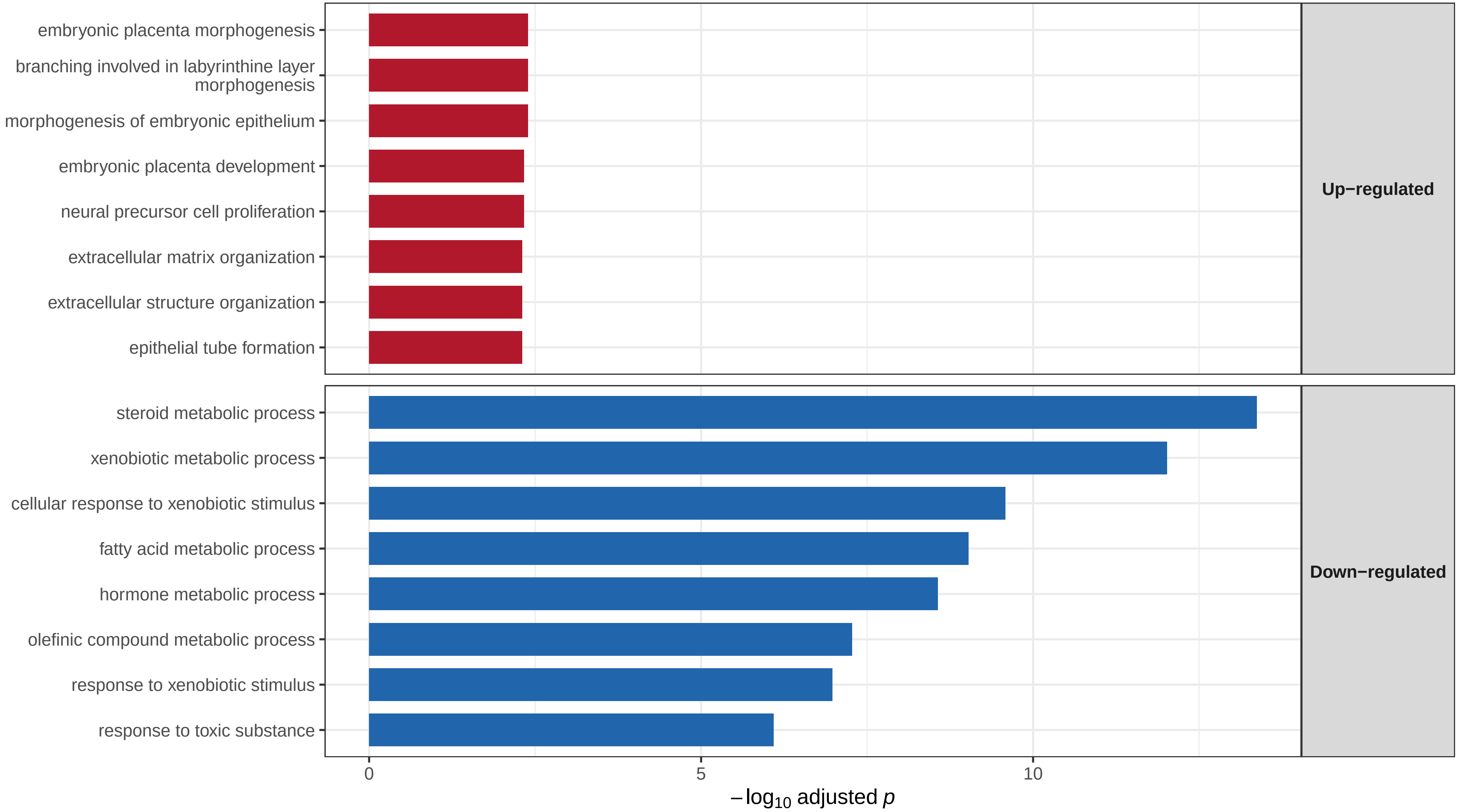

Supplement: Supplementary file 1 [file biology-15-01287-s001.zip › Figure_S3_GO_C3.pdf]
