# Supplementary material for: A Bioinformatics Analysis Based on Omics and Clinical Data for Graph-Based Patient Stratification in Hepatocellular Carcinoma
Source: Biology (Basel). 2026 Aug 4;15(15):1287. doi: 10.3390/biology15151287 (PMC13465779; doi:10.3390/biology15151287)

Figure S5. Subtype C5 − Cholangio−mesenchymal

GO Biological Process enrichment (up− vs down−regulated genes)

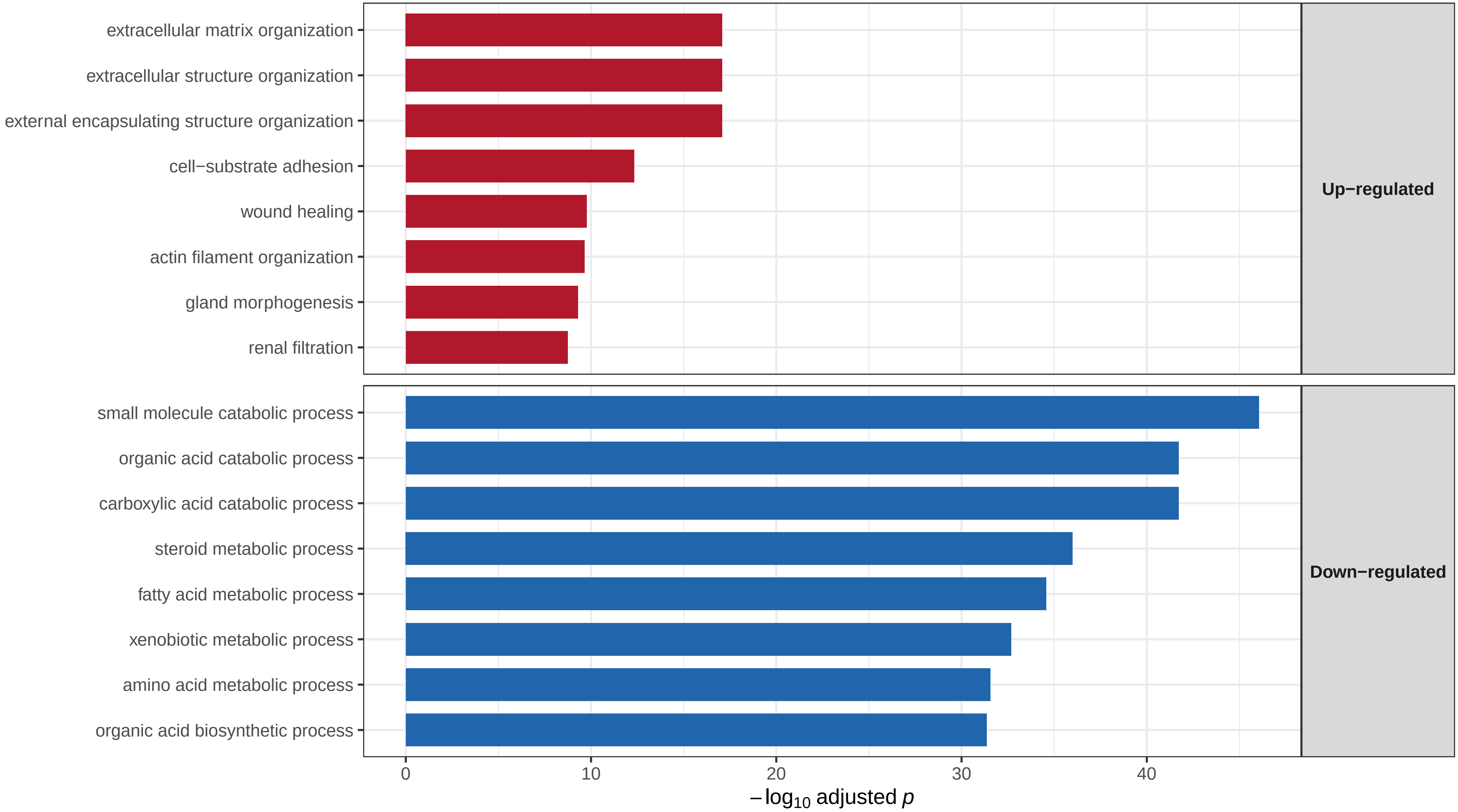

Supplement: Supplementary file 1 [file biology-15-01287-s001.zip › Figure_S5_GO_C5.pdf]
