# Supplementary material for: A Bioinformatics Analysis Based on Omics and Clinical Data for Graph-Based Patient Stratification in Hepatocellular Carcinoma
Source: Biology (Basel). 2026 Aug 4;15(15):1287. doi: 10.3390/biology15151287 (PMC13465779; doi:10.3390/biology15151287)
Supplement: Supplementary file 1 [file biology-15-01287-s001.zip › Figure_S6_GO_C6.pdf]

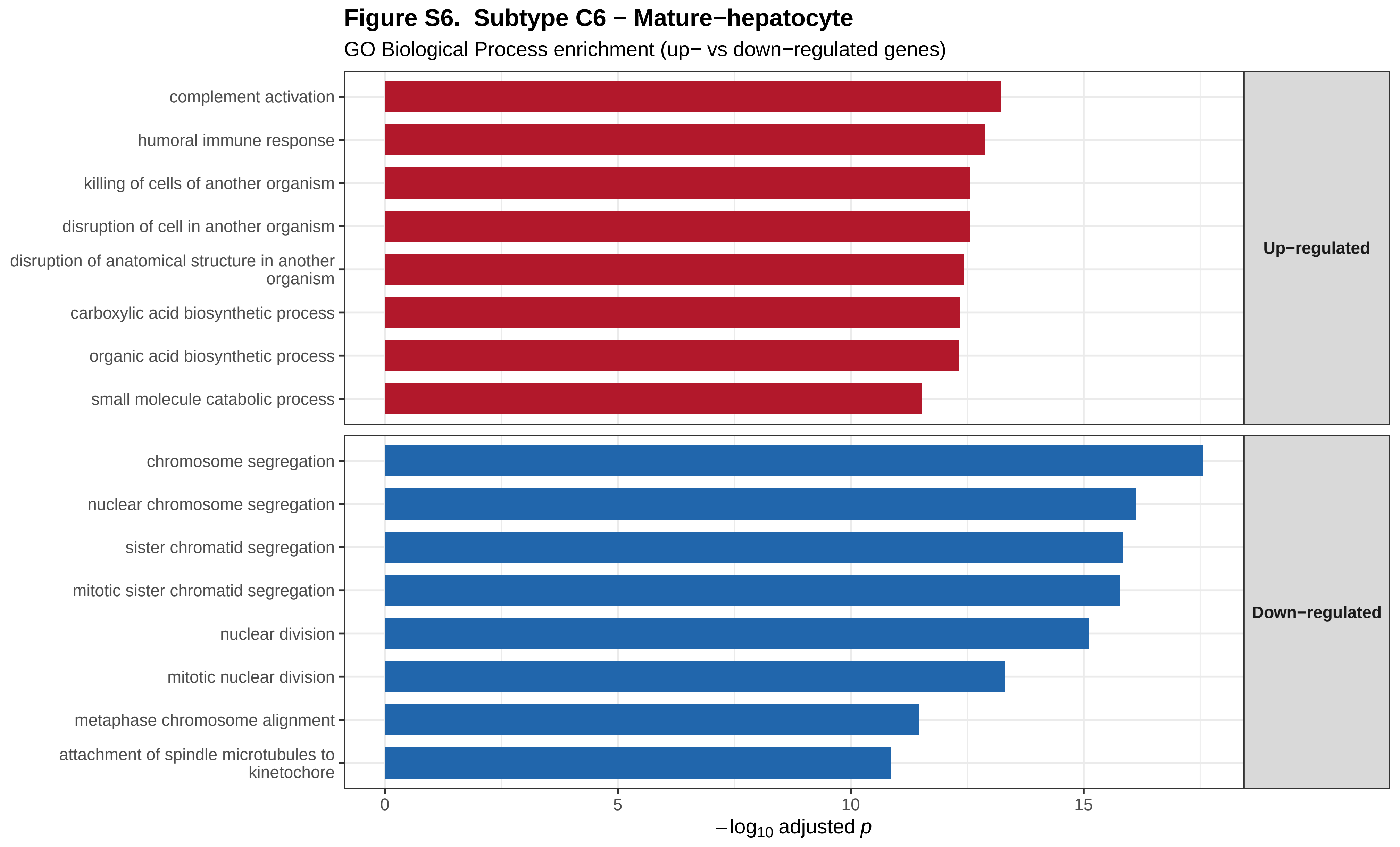
Figure S6. Subtype C6 − Mature−hepatocyte
GO Biological Process enrichment (up− vs down−regulated genes)
complement activation
humoral immune response
killing of cells of another organism
disruption of cell in another organism
disruption of anatomical structure in another organism
carboxylic acid biosynthetic process
organic acid biosynthetic process
small molecule catabolic process
Up−regulated
chromosome segregation
nuclear chromosome segregation
sister chromatid segregation
mitotic sister chromatid segregation
nuclear division
mitotic nuclear division
metaphase chromosome alignment
attachment of spindle microtubules to kinetochore
Down−regulated
0
5
10
15
−log10 adjusted p
