# Supplementary material for: A Bioinformatics Analysis Based on Omics and Clinical Data for Graph-Based Patient Stratification in Hepatocellular Carcinoma
Source: Biology (Basel). 2026 Aug 4;15(15):1287. doi: 10.3390/biology15151287 (PMC13465779; doi:10.3390/biology15151287)
Supplement: Supplementary file 1 [file biology-15-01287-s001.zip › Figure_S7_purity_immune.pdf]

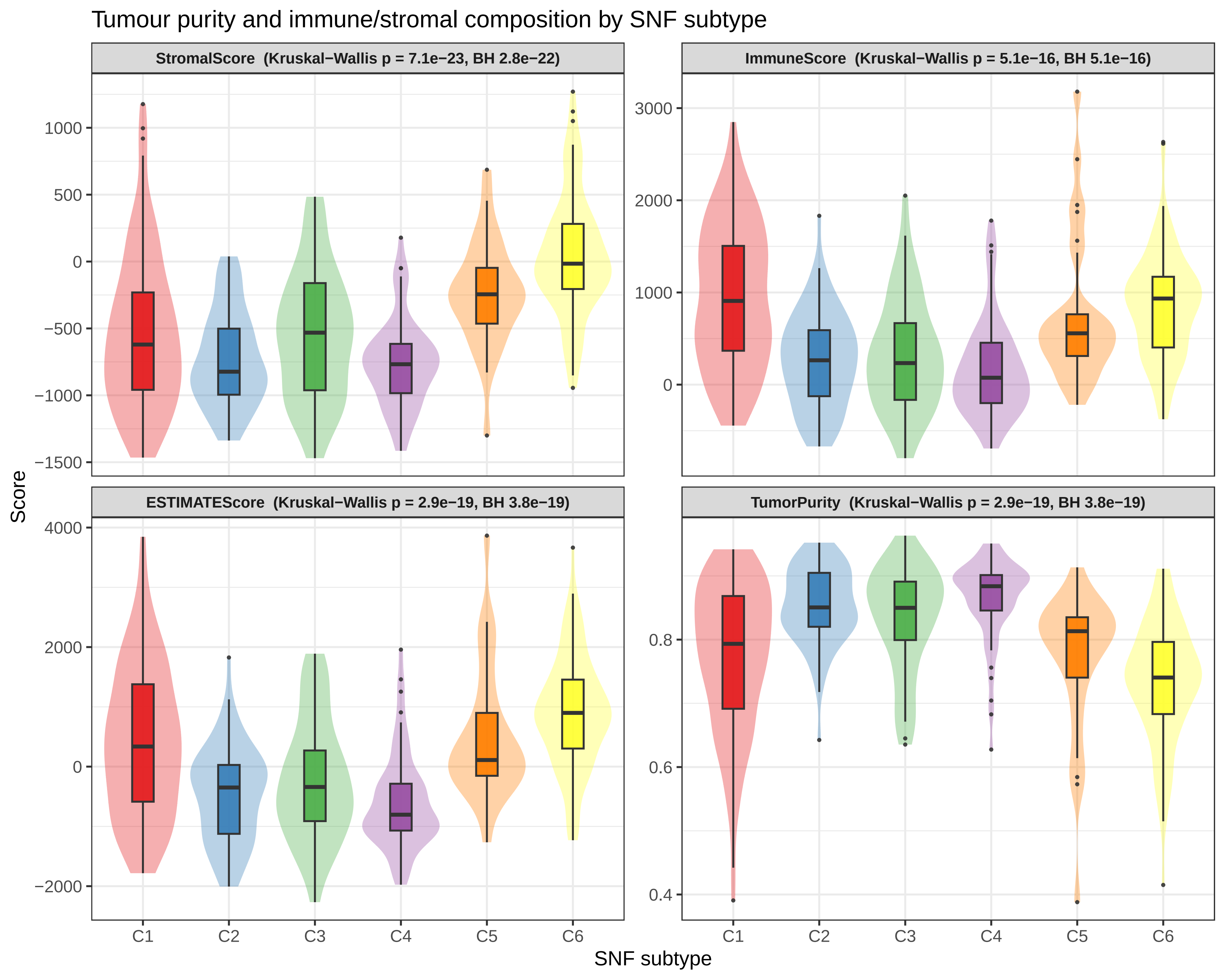

Tumour purity and immune/stromal composition by SNF subtype
StromalScore (Kruskal−Wallis p = 7.1e−23, BH 2.8e−22)
ImmuneScore (Kruskal−Wallis p = 5.1e−16, BH 5.1e−16)
ESTIMATEScore (Kruskal−Wallis p = 2.9e−19, BH 3.8e−19)
TumorPurity (Kruskal−Wallis p = 2.9e−19, BH 3.8e−19)
Score
SNF subtype
C1
C2
C3
C4
C5
C6
1000
500
0
−500
−1000
−1500
3000
2000
1000
0
4000
2000
0
−2000
0.8
0.6
0.4
