# Supplementary material for: A Bioinformatics Analysis Based on Omics and Clinical Data for Graph-Based Patient Stratification in Hepatocellular Carcinoma
Source: Biology (Basel). 2026 Aug 4;15(15):1287. doi: 10.3390/biology15151287 (PMC13465779; doi:10.3390/biology15151287)
Supplement: Supplementary file 1 [file biology-15-01287-s001.zip › Table_S1.pdf]

Table S1.

Stability of the survival separation across SNF hyper-parameters (K, T) and cluster number. For each setting: number of patients (N), log-rank chi-square, degrees of freedom, log-rank p-value, and Adjusted Rand Index versus the published six-subtype partition. The selected configuration (K = 10, T = 20, n = 6) is highlighted.

K	T	n clusters	N	Log-rank χ^2	df	Log-rank p	ARI vs published
8	15	5	359	12.626	4	1.33e-02	0.598
10	15	5	359	10.874	4	2.80e-02	0.609
12	15	5	359	9.680	4	4.62e-02	0.565
8	20	5	359	15.470	4	3.82e-03	0.588
10	20	5	359	8.837	4	6.53e-02	0.622
12	20	5	359	9.403	4	5.18e-02	0.577
8	25	5	359	11.557	4	2.10e-02	0.595
10	25	5	359	12.983	4	1.14e-02	0.615
12	25	5	359	8.537	4	7.38e-02	0.575
8	15	6	359	18.853	5	2.05e-03	0.844
10	15	6	359	19.209	5	1.76e-03	0.937
12	15	6	359	16.637	5	5.24e-03	0.868
8	20	6	359	23.182	5	3.12e-04	0.851
10	20	6	359	22.828	5	3.64e-04	1.000
12	20	6	359	24.587	5	1.67e-04	0.920
8	25	6	359	22.785	5	3.71e-04	0.812
10	25	6	359	21.690	5	5.99e-04	0.923
12	25	6	359	24.495	5	1.74e-04	0.906
8	15	7	359	34.791	6	4.73e-06	0.610
10	15	7	359	22.295	6	1.07e-03	0.642
12	15	7	359	25.886	6	2.34e-04	0.640
8	20	7	359	35.571	6	3.34e-06	0.610
10	20	7	359	26.223	6	2.02e-04	0.651
12	20	7	359	24.146	6	4.91e-04	0.638
8	25	7	359	33.192	6	9.63e-06	0.602
10	25	7	359	31.457	6	2.07e-05	0.631
12	25	7	359	29.134	6	5.74e-05	0.658
