# Supplementary material for: A Bioinformatics Analysis Based on Omics and Clinical Data for Graph-Based Patient Stratification in Hepatocellular Carcinoma
Source: Biology (Basel). 2026 Aug 4;15(15):1287. doi: 10.3390/biology15151287 (PMC13465779; doi:10.3390/biology15151287)
Supplement: Supplementary file 1 [file biology-15-01287-s001.zip › Table_S1b_cluster_k.pdf]

Table S1b. Selection of the number of clusters. Eigen-gap of the normalised graph Laplacian, average silhouette width, the four components of the composite quality score (mean silhouette, separation ratio scaled to its maximum across the grid, Normalised Mutual Information between the individual omics networks, and cluster balance) and the resulting composite score $Q = 0.4 \cdot s + 0.3 \cdot (r/r\_max) + 0.2 \cdot m + 0.1 \cdot b$, computed on the final fused SNF network ($K = 10$, $\sigma = 1$, $T = 20$) for $k = 2$–10. The adopted six-cluster solution is highlighted.

k	Eigen-gap	Mean silhouette	Separation ratio (norm.)	NMI	Cluster balance	Composite score Q
**2**	0.0544	0.00134	0.385	0.004	0.956	0.212
**3**	0.0268	0.00170	0.479	0.110	0.770	0.243
**4**	0.0559	0.00199	0.703	0.165	0.152	0.260
**5**	0.0404	0.00235	0.812	0.175	0.000	0.280
**6**	**0.0326**	**0.00272**	**0.848**	**0.220**	**0.272**	**0.327**
**7**	0.0406	0.00308	0.913	0.226	0.375	0.358
**8**	0.0655	0.00328	0.917	0.275	0.545	0.386
**9**	0.0054	0.00343	0.998	0.321	0.506	0.415
**10**	0.0293	0.00363	1.000	0.330	0.535	0.421

Note. None of the three internal criteria identifies k = 6 as an optimum: the eigen-gap is maximal at k = 8 (second-highest at k = 4; SNFtool::estimateNumberOfClustersGivenGraph returns 8 and 4 as the two best estimates), while the average silhouette width and the composite score both increase monotonically up to k = 10. The six-cluster solution was therefore not selected on the basis of a sharp internal optimum but retained for prognostic and biological interpretability and parsimony (see Section 2.4 and Discussion).
