# Supplementary material for: A Bioinformatics Analysis Based on Omics and Clinical Data for Graph-Based Patient Stratification in Hepatocellular Carcinoma
Source: Biology (Basel). 2026 Aug 4;15(15):1287. doi: 10.3390/biology15151287 (PMC13465779; doi:10.3390/biology15151287)
Supplement: Supplementary file 1 [file biology-15-01287-s001.zip › Table_S2.pdf]

Table S2.

Sensitivity of the subtypes and their survival separation to symmetric, IQR-only feature selection applied identically to mRNA and miRNA (no paired differential expression). ARI and log-rank p-value are computed against the published six-subtype solution.

Property	Value
**Feature-selection scheme**	symmetric IQR (no paired DE); same percentile mRNA & miRNA
**IQR percentile threshold**	0.64
**mRNA features selected**	20329
**miRNA features selected**	307
**Patients**	366
**Clusters obtained**	6
**ARI vs published partition**	0.674
**Log-rank chi-square**	18.696
**Log-rank df**	5
**Log-rank p-value**	2.19e-03
