# Supplementary material for: A Bioinformatics Analysis Based on Omics and Clinical Data for Graph-Based Patient Stratification in Hepatocellular Carcinoma
Source: Biology (Basel). 2026 Aug 4;15(15):1287. doi: 10.3390/biology15151287 (PMC13465779; doi:10.3390/biology15151287)
Supplement: Supplementary file 1 [file biology-15-01287-s001.zip › Table_S3.pdf]

Table S3.

Kruskal-Wallis comparison of ESTIMATE stromal, immune and ESTIMATE scores and Aran tumour purity across the six subtypes (Benjamini-Hochberg adjusted), with per-subtype median (interquartile range).

Score	χ^2	df	p	p (BH)	C1	C2	C3	C4	C5	C6
StromalScore	113.60	5	7.11e-23	2.84e-22	-620.4 (728.0)	-823.3 (493.6)	-531.7 (801.2)	-767.6 (368.5)	-244.8 (417.0)	-15.8 (487.0)
ImmuneScore	81.03	5	5.12e-16	5.12e-16	908.2 (1137.5)	264.0 (716.2)	233.6 (832.8)	75.0 (654.5)	556.7 (452.2)	934.2 (767.1)
ESTIMATEScore	96.52	5	2.86e-19	3.82e-19	336.5 (1964.7)	-349.3 (1152.2)	-340.6 (1181.8)	-804.1 (783.6)	110.4 (1051.8)	899.2 (1153.1)
TumorPurity	96.52	5	2.86e-19	3.82e-19	0.8 (0.2)	0.9 (0.1)	0.8 (0.1)	0.9 (0.1)	0.8 (0.1)	0.7 (0.1)
