# Supplementary material for: A Bioinformatics Analysis Based on Omics and Clinical Data for Graph-Based Patient Stratification in Hepatocellular Carcinoma
Source: Biology (Basel). 2026 Aug 4;15(15):1287. doi: 10.3390/biology15151287 (PMC13465779; doi:10.3390/biology15151287)
Supplement: Supplementary file 1 [file biology-15-01287-s001.zip › Table_S3b.pdf]

Table S3b.
Dunn's post-hoc pairwise comparisons between subtypes (Benjamini-Hochberg adjusted) for each of the four ESTIMATE-derived scores.

Score	Comparison	Z	p	p (BH)
ESTIMATEScore	C1-C2	4.55	5.25e-06	1.57e-05
ESTIMATEScore	C1-C3	2.92	3.56e-03	5.93e-03
ESTIMATEScore	C1-C4	5.30	1.18e-07	4.41e-07
ESTIMATEScore	C1-C5	-0.47	6.35e-01	6.35e-01
ESTIMATEScore	C1-C6	-3.16	1.57e-03	2.95e-03
ESTIMATEScore	C2-C3	-1.07	2.85e-01	3.29e-01
ESTIMATEScore	C2-C4	0.88	3.80e-01	4.07e-01
ESTIMATEScore	C2-C5	-3.65	2.60e-04	5.56e-04
ESTIMATEScore	C2-C6	-7.41	1.25e-13	9.41e-13
ESTIMATEScore	C3-C4	1.83	6.69e-02	9.12e-02
ESTIMATEScore	C3-C5	-2.59	9.48e-03	1.42e-02
ESTIMATEScore	C3-C6	-5.49	4.11e-08	2.06e-07
ESTIMATEScore	C4-C5	-4.25	2.19e-05	5.47e-05
ESTIMATEScore	C4-C6	-8.03	9.90e-16	1.48e-14
ESTIMATEScore	C5-C6	-1.68	9.39e-02	1.17e-01
ImmuneScore	C1-C2	5.59	2.27e-08	1.14e-07
ImmuneScore	C1-C3	4.62	3.79e-06	1.14e-05
ImmuneScore	C1-C4	6.60	4.12e-11	6.18e-10
ImmuneScore	C1-C5	1.24	2.14e-01	2.92e-01
ImmuneScore	C1-C6	0.08	9.33e-01	9.33e-01
ImmuneScore	C2-C3	-0.32	7.47e-01	8.00e-01
ImmuneScore	C2-C4	1.17	2.42e-01	2.79e-01
ImmuneScore	C2-C5	-2.73	6.43e-03	1.21e-02
ImmuneScore	C2-C6	-5.43	5.70e-08	2.14e-07
ImmuneScore	C3-C4	1.37	1.69e-01	2.54e-01
ImmuneScore	C3-C5	-2.31	2.10e-02	3.50e-02
ImmuneScore	C3-C6	-4.50	6.85e-06	1.71e-05
ImmuneScore	C4-C5	-3.56	3.71e-04	7.95e-04
ImmuneScore	C4-C6	-6.43	1.25e-10	9.35e-10
ImmuneScore	C5-C6	-1.18	2.40e-01	3.00e-01
StromalScore	C1-C2	2.13	3.33e-02	4.99e-02
StromalScore	C1-C3	-0.09	9.25e-01	9.25e-01
StromalScore	C1-C4	2.34	1.93e-02	3.21e-02
StromalScore	C1-C5	-2.89	3.86e-03	8.28e-03
StromalScore	C1-C6	-7.06	1.71e-12	8.54e-12
StromalScore	C2-C3	-1.88	6.04e-02	6.97e-02
StromalScore	C2-C4	0.28	7.77e-01	8.33e-01

Score	Comparison	Z	p	p (BH)
StromalScore	C2-C5	-4.28	1.91e-05	4.77e-05
StromalScore	C2-C6	-8.63	6.36e-18	9.54e-17
StromalScore	C3-C4	2.08	3.78e-02	5.16e-02
StromalScore	C3-C5	-2.54	1.09e-02	2.05e-02
StromalScore	C3-C6	-5.72	1.04e-08	3.90e-08
StromalScore	C4-C5	-4.40	1.09e-05	3.26e-05
StromalScore	C4-C6	-8.57	1.06e-17	7.94e-17
StromalScore	C5-C6	-1.92	5.42e-02	6.77e-02
TumorPurity	C1-C2	-4.55	5.25e-06	1.57e-05
TumorPurity	C1-C3	-2.92	3.56e-03	5.93e-03
TumorPurity	C1-C4	-5.30	1.18e-07	4.41e-07
TumorPurity	C1-C5	0.47	6.35e-01	6.35e-01
TumorPurity	C1-C6	3.16	1.57e-03	2.95e-03
TumorPurity	C2-C3	1.07	2.85e-01	3.29e-01
TumorPurity	C2-C4	-0.88	3.80e-01	4.07e-01
TumorPurity	C2-C5	3.65	2.60e-04	5.56e-04
TumorPurity	C2-C6	7.41	1.25e-13	9.41e-13
TumorPurity	C3-C4	-1.83	6.69e-02	9.12e-02
TumorPurity	C3-C5	2.59	9.48e-03	1.42e-02
TumorPurity	C3-C6	5.49	4.11e-08	2.06e-07
TumorPurity	C4-C5	4.25	2.19e-05	5.47e-05
TumorPurity	C4-C6	8.03	9.90e-16	1.48e-14
TumorPurity	C5-C6	1.68	9.39e-02	1.17e-01
