# Supplementary material for: A Bioinformatics Analysis Based on Omics and Clinical Data for Graph-Based Patient Stratification in Hepatocellular Carcinoma
Source: Biology (Basel). 2026 Aug 4;15(15):1287. doi: 10.3390/biology15151287 (PMC13465779; doi:10.3390/biology15151287)
Supplement: Supplementary file 1 [file biology-15-01287-s001.zip › Table_S4.pdf]

## Table S4.

*Benchmarking of the six subtypes against published HCC transcriptional classifications and TP53/CTNNB1 mutation status; association by Fisher's exact or Monte-Carlo Fisher test (Benjamini-Hochberg adjusted). Schemes whose signature could not be retrieved are marked not available (shaded).*

| Scheme | Availability | Gene source | Genes | Patients | Classes | Test | p | p (BH) |
|---|---|---|---|---|---|---|---|---|
| Hoshida | AVAILABLE | *Hoshida et al., Cancer Res 2009 (MSigDB C2:CGP)* | 616 | 366 | 3 | Fisher (Monte-Carlo, B=1e5) | 1.00e-05 | 1.00e-05 |
| Boyault | AVAILABLE | *Boyault et al., Hepatology 2007 (MSigDB C2:CGP; atomic subgroup G1/G3/G6 UP signatures, not the 16-gene predictor)* | 371 | 366 | 3 | Fisher (Monte-Carlo, B=1e5) | 1.00e-05 | 1.00e-05 |
| Wnt/CTNNB1 | AVAILABLE | *CHIANG_LIVER_CANCER_SUBCLASS_CTNNB1_UP — Chiang et al., Cancer Res 2008 (MSigDB C2:CGP)* | 175 | 366 | 2 | Fisher exact | 3.47e-23 | 2.43e-22 |
| Immune | AVAILABLE | *HALLMARK_INFLAMMATORY_RESPONSE — MSigDB Hallmark (Liberzon et al. 2015); immune-activation proxy, NOT the Sia 2017 immune class* | 200 | 366 | 2 | Fisher exact | 2.16e-19 | 7.57e-19 |
| Progenitor | AVAILABLE | *YAMASHITA_LIVER_CANCER_STEM_CELL_UP — Yamashita et al. 2008 (MSigDB C2:CGP)* | 47 | 366 | 2 | Fisher exact | 5.04e-17 | 8.82e-17 |
| iCluster | NOT AVAILABLE | *no sample->iCluster mapping under data/signatures/* | | | | | n/a | n/a |
| Mutation: TP53 | AVAILABLE | *GDC TCGA-LIHC Masked Somatic Mutation (TCGAbiolinks)* | 1 | 366 | 2 | Fisher exact | 4.51e-08 | 6.31e-08 |
| Mutation: CTNNB1 | AVAILABLE | *GDC TCGA-LIHC Masked Somatic Mutation (TCGAbiolinks)* | 1 | 366 | 2 | Fisher exact | 1.40e-17 | 3.27e-17 |
