# Supplementary material for: A Bioinformatics Analysis Based on Omics and Clinical Data for Graph-Based Patient Stratification in Hepatocellular Carcinoma
Source: Biology (Basel). 2026 Aug 4;15(15):1287. doi: 10.3390/biology15151287 (PMC13465779; doi:10.3390/biology15151287)
Supplement: Supplementary file 1 [file biology-15-01287-s001.zip › Table_S5.pdf]

Table S5. External-validation sensitivity analysis in GSE14520:

full (299-gene) versus array-restricted (293-gene) centroid signature

Metric	Full signature	Array-restricted signature
Signature size (genes)	299	293
Genes present on GPL3921 array	174 (58.2%)	293 (100%)
Tumours classified	225	225
Tumours with OS follow-up / events	221 / 85	221 / 85
Six-subtype log-rank p-value	0.011	0.018
C1 vs rest — HR (95% CI)	2.20 (1.37–3.54)	2.15 (1.32–3.50)
C1 vs rest — p-value	0.0011	0.0021
Multivariate cohort — N / events	219 / 84	219 / 84
C4 vs C1 — multivariate HR	0.365	0.378
C4 vs C1 — multivariate p-value	0.012	0.016
Spearman/Euclidean agreement	85.8%	85.3%
Label concordance with full signature	— (reference)	88.9%

Restricting the signature a priori to markers present on the HT_HG-U133A array (GPL3921) removes the cross-platform gene-overlap objection (58.2% → 100% coverage) while reproducing every survival statistic. HR, hazard ratio; CI, confidence interval; OS, overall survival. C1 = Proliferative–oncofetal; C4 = Hepatocyte-zonation-like.
