# Supplementary material for: A Bioinformatics Analysis Based on Omics and Clinical Data for Graph-Based Patient Stratification in Hepatocellular Carcinoma
Source: Biology (Basel). 2026 Aug 4;15(15):1287. doi: 10.3390/biology15151287 (PMC13465779; doi:10.3390/biology15151287)
Supplement: Supplementary file 1 [file biology-15-01287-s001.zip › Table_S6.pdf]

Supplementary Table S6. Numerical summary of the cluster robustness analysis: subsampling ARI, per-subtype co-clustering consensus, hyper-parameter (K/T) sensitivity, and subsampling stability across candidate cluster numbers (k = 2–10).

Global summary

Metric	Value
N resamples	100
Subsample fraction	0.80
ARI mean	0.839
ARI median	0.840
ARI min	0.760
ARI SD	0.008
Overall within-cluster consensus	0.77

Per-subtype within-cluster consensus

Subtype	Within-cluster consensus
C1 Proliferative–oncofetal	0.72
C2 Xenobiotic-metabolizing	0.80
C3 Imprinted/fetal-reactivated	0.69
C4 Hepatocyte-zonation-like	0.73
C5 Cholangio-mesenchymal	0.94
C6 Mature-hepatocyte/quiescent	0.75

Hyper-parameter sensitivity (ARI vs reference)

K	ARI	T	ARI
6	0.693	10	0.821
8	0.851	15	0.937
10	1.000	20	1.000
12	0.920	25	0.923
15	0.852	30	0.924
20	0.741	50	0.871
25	0.561		
30	0.548		

Stability across-k

k	Mean ARI (100 subsamples, 80%)	SD
2	0.676	0.002
3	0.845	0.058
4	0.722	0.009
5	0.571	0.012
6	0.839	0.015
7	0.718	0.004
8	0.786	0.001
9	0.701	0.004
10	0.794	0.003

Values in the across-k panel derive from an independent resampling run; small Monte-Carlo differences from the k = 6 summary above are expected.
